# Identification of immune and stromal cell infiltration-related gene signature for prognosis prediction in acute lymphoblastic leukemia

**DOI:** 10.18632/aging.204292

**Published:** 2022-09-19

**Authors:** Wen-Liang Yu, Zi-Chun Hua

**Affiliations:** 1 School of Biopharmacy, China Pharmaceutical University, Nanjing 211198, China; 2 Changzhou High-Tech Research Institute of Nanjing University and Jiangsu TargetPharma Laboratories Inc., Changzhou 213164, China; 3 School of Life Sciences, Nanjing University, Nanjing 210023, China

**Keywords:** acute lymphoblastic leukemia, immune cell infiltration, overall survival, bone marrow microenvironment, bioinformatics

## Abstract

Acute lymphoblastic leukemia (ALL) is a common and life-threatening hematologic malignancy, its occurrence and progression are closely related to immune/stromal cell infiltration in the bone marrow (BM) microenvironment. However, no studies have described an immune/stromal cell infiltration-related gene (ISCIRG)-based prognostic signature for ALL. A total of 444 patients involving 437 bulk and 7 single-cell RNA-seq datasets were included in this study. Eligible datasets were searched and reviewed from the database of TCGA, TARGET project and GEO. Then an integrated bioinformatics analysis was performed to select optimal prognosis-related genes from ISCIRGs, construct a nomogram model for predicting prognosis, and assess the predictive power. After LASSO and multivariate Cox regression analyses, a seven ISCIRGs-based signature was proved to be able to significantly stratify patients into high- and low-risk groups in terms of OS. The seven genes were confirmed that directly related to the composition and status of immune/stromal cells in BM microenvironment by analyzing bulk and single-cell RNA-seq datasets. The calibration plot showed that the predicted results of the nomogram were consistent with the actual observation results of training/validation cohort. This study offers a reference for future research regarding the role of ISCIRGs in ALL and the clinical care of patients.

## INTRODUCTION

Acute lymphoblastic leukemia (ALL) is one of the most frequent hematologic malignancies, especially in children, accounting for approximately 25% of all pediatric cancers [1]. The immunophenotypes of ALL include T cell ALL (T-ALL), B cell ALL (B-ALL) and mixed-phenotype acute leukemia (MPAL), where MPAL is a rare immunophenotype with features of both ALL and acute myeloid leukemia (AML) [2]. Although the application of immunotherapy led by chimeric antigen receptor T (CAR T) cell therapy has greatly improved the clinical remission rate of ALL patients [3], a shorter overall survival (OS) rate caused by adverse prognosis is still a crucial challenge for clinicians and patients. For example, the OS rate of adult ALL patients is less than 45% [4].

Accumulating evidence indicates that the crosstalk between tumor and immune cells plays a crucial role in cancer development by regulating tumor malignancy, immune/stromal cell infiltration, and immune evasion in the tumor microenvironment [5–8]. Similar to solid tumors, the bone marrow (BM) microenvironment of ALL is a dynamic system of immune cells, endothelial progenitor cells, stromal cells, extracellular matrix, growth factors and cytokines. Therefore, evaluating the role of immune/stromal cell infiltration of the BM microenvironment in survival and progression and identifying novel accurate biomarkers for assessing the immune/stromal cell infiltration-related risk in patients with ALL is of utmost importance for improving the prognosis.

Previous studies confirmed that activated stromal cells could rescue ALL cells from oxidative stress by transferring mitochondria [9], and some immune cells could predict outcomes in ALL patients [10]. For example, the proportion of PD1+TIM3+ double-positive CD4+ T cells could predict poor survival in adult B-ALL patients [10, 11], and increased frequencies of activated cytokine-producing natural killer (NK) cells could independently predict poor clinical outcome in ALL patient [12]. In tumor microenvironment, cytokines played a major role in the regulation of the cellular responses between tumor cells and immune cells, for example, TGFβ, IL-10 and IL-6 could dampen the anti-tumor response of NK cells by suppressing activity and promote subsequent tumor evasion and progression [13, 14].

The Estimation of Stromal and Immune cells in Malignant Tumors using Expression data (ESTIMATE) algorithm is an analysis approach based on single sample gene expression signatures to infer the fraction of stromal and immune cells and to generate immune/stromal scores for predicting the infiltration of stromal and immune cells in malignant tumors [15]. It has made outstanding progress in a variety of solid malignancies and some hematological malignancies, such as glioma [16], prostate cancer [17], gastric cancer [18], colon cancer [19] and AML [20]. At present, the prognosis prediction models regarding ALL are still based on specific genes or some clinicopathologic characteristics [21–23]. Therefore, this study aims to investigate the infiltration of immune/stromal cells in the BM microenvironment and construct an accurate immune/stromal cell infiltration-related genes (ISCIRGs)-based model for prognosis prediction of ALL patients.

## RESULTS

### Data source and clinicopathologic characteristics of patients

**Figure 1:**
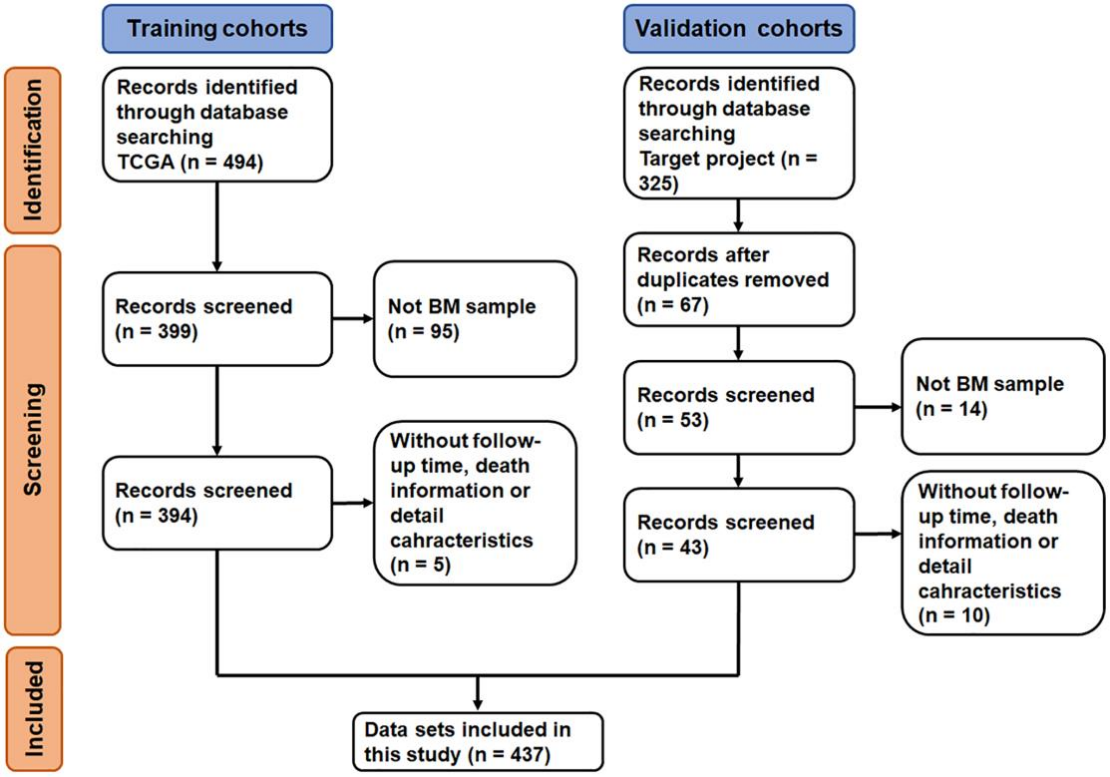
Flow-diagram of the datasets selection process.

The datasets selection process is shown in Figure 1. By reviewing the information of ALL related datasets from The Cancer Genome Atlas (TCGA) and the Therapeutically Applicable Research to Generate Effective Treatments (TARGET) project database, 494 and 325 primary datasets were identified for training group and validation group, respectively. After eliminating 95 datasets that were not derived from BM samples and 5 datasets without follow-up time, survival information and detailed clinical information, 394 datasets were included in training group. Meanwhile, after removing duplicates, 14 datasets that were not derived from BM samples and 10 datasets without clinical information (i.e., follow-up time, survival information, sex, age, and race), 43 datasets were included in validation group. Subsequently, the complete gene expression profiles and the corresponding metadata and clinical profiles were downloaded and merged.

A total of 437 ALL patients, including 288 (65.9%) males and 140 (34.1%) females, were finally included in this study (Figure 1). The age at initial pathological diagnosis ranged from 1 to 30 years, including 418 (95.7%) children (<18 years old) and 19 (4.3%) adults (≥18 years old). The three primary diagnosis immunophenotypes were T-ALL (242, 55.4%), B-ALL (147, 33.6%) and MPAL (7, 1.6%). The ethnicity included Asian (21, 4.8%), black or African American (43, 9.6%), native Hawaiian or other Pacific Islander (3, 0.7%) and White (294, 67.3%); 76 patients (17.4%) were unknown. There were no significant statistical differences between the two groups of characteristic variables (*p* > 0.05, Table 1, Supplementary Table 1).

**Table 1:** Demographics and clinicopathologic characteristics of patients with ALL.

**Demographic or characteristic**	**Training cohort (*n* = 394)**	**Validation cohort (*n* = 43)**	*p* ^*^	**Total (*n* = 437)**
**Gender, No. (%)**				
Male	265 (67.3)	23 (53.5)	0.070	288 (65.9)
Female	129 (32.7)	20 (46.5)		149 (34.1)
**Age (years), No. (%)**				
<18	375 (95.2)	43 (100)	0.141	418 (95.7)
≥18	19 (4.8)	0 (0)		19 (4.3)
**Immunophenotypes, No. (%)**				
B-ALL	145 (36.8)	2 (4.7)	−	147 (33.6)
T-ALL	242 (61.4)	−		242 (55.4)
MPAL	7 (1.8)	−		7 (1.6)
Unknown	0 (0)	41 (95.3)		41 (9.4)
**Ethnicity, No. (%)**				
Asian	19 (4.8)	2 (4.7)	−	21 (4.8)
Black or African American, No. (%)	43 (10.9)	−		43 (9.8)
Native Hawaiian or other Pacific Islander	3 (0.8)	−		3 (0.7)
White	287 (72.8)	7 (16.3)		294 (67.3)
Unknown	42 (10.7)	34 (79)		76 (17.4)

^*^p value was calculated by Pearson chi-square test.

### Identification of immune and stromal cell infiltration in training cohort

**Figure 2:**
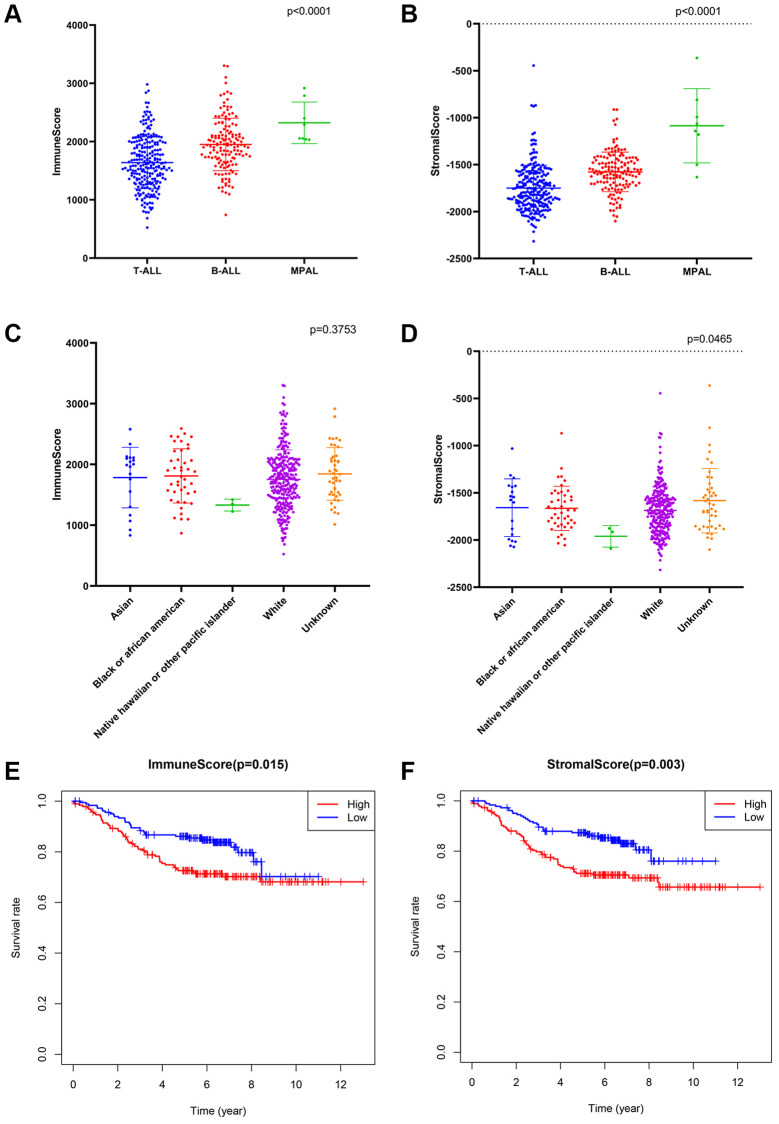
**The association between immune conditions and the clinical features in training group.** (**A**, **B**) Distribution of immune scores and stromal scores for immunophenotypes of ALL patients. (**C**, **D**) Distribution of immune score and stromal score for races of ALL patients. (**E**) KM survival curve for comparison between samples with high and low stromal scores. (**F**) KM survival curve for comparison between samples with high and low immune scores.

The ESTIMATE algorithm was applied to calculate the immune/stromal score for all included samples, in training cohort, the former ranged from 524.82 to 3304.62, and the latter ranged from −2316.13 to −362.69 (Supplementary Table 1). The immune score had a significant association with immunophenotype (*p* < 0.0001, Figure 2A) but not with race (*p* = 0.3753, Figure 2C), and the stromal scores were significantly associated with both the immunophenotype (*p* < 0.0001, Figure 2B) and race (*p* = 0.0465, Figure 2D).

Subsequently, the included ALL patients were classified into high (*n* = 197) and low score (*n* = 197) groups to explore the potential relationships in OS vs. immune scores and OS vs. stromal scores. Kaplan–Meier (KM) survival analysis showed that the OS time of patients in both the high immune score group and the high stromal score group was significantly shorter than that of patients in the low immune score group (*p* = 0.015, Figure 2E) and the low stromal score group (*p* = 0.003) (*p* > 0.05, Figure 2F). The above findings suggest that the OS of ALL patients is significantly associated with both the immune score and stromal score.

### Identification of common differentially expressed genes (DEGs) based on immune/stromal scores

**Figure 3:**
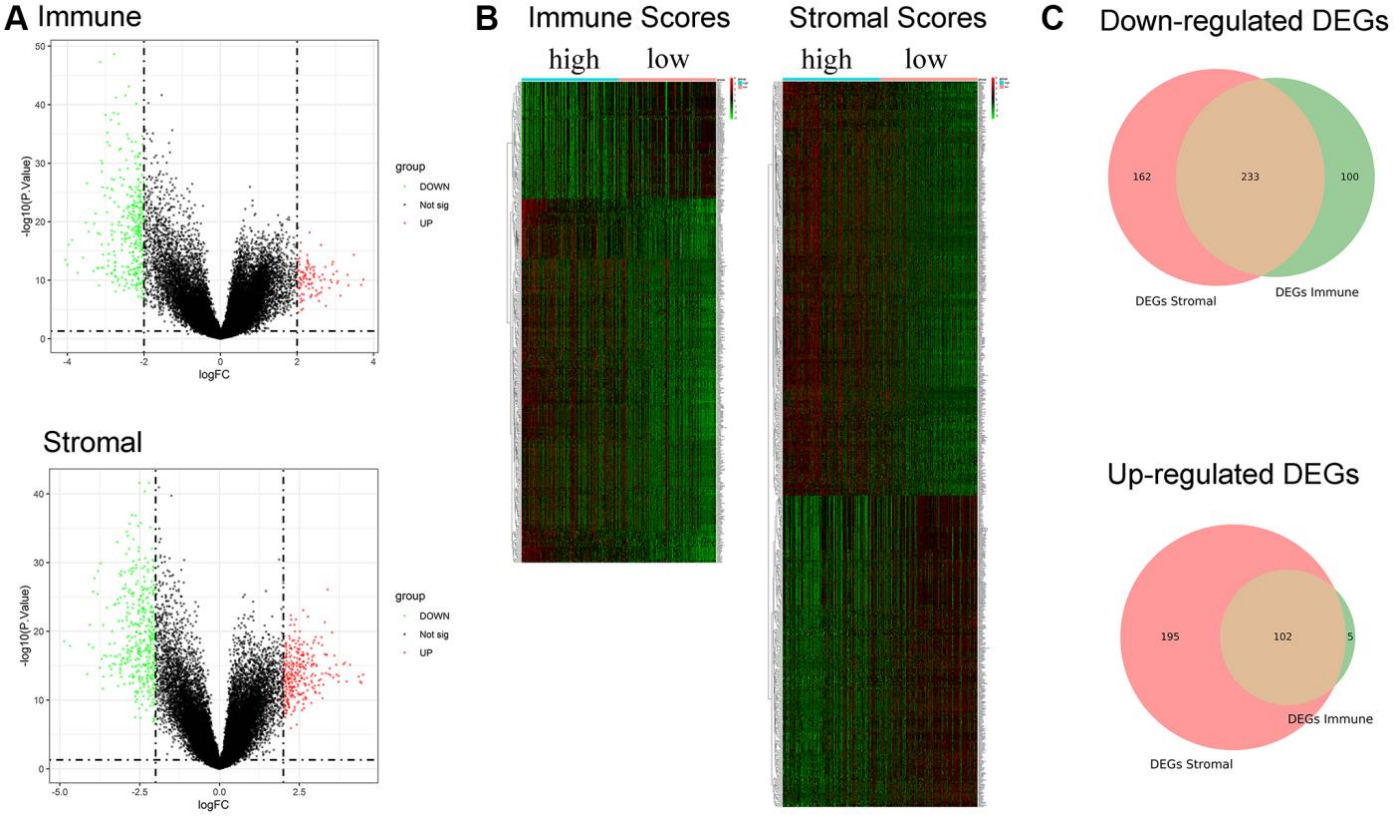
**Identification of DEGs based on immune score and stromal score.** (**A**) Volcano plot of DEGs from the low/high immune and stromal score groups. Note: Genes with *p* < 0.05 are shown in red (FC > 2) and green (FC < −2). Black plots represent the remaining genes (those with no significant difference). (**B**) Heatmap of DEGs from the low/high immune and stromal score groups. (**C**) Venn plot for common up- and downregulated DEGs in the stromal and immune score groups.

A total of 440 and 692 DEGs between high/low immune and stromal scores were identified, respectively (Figure 3A). The heatmap is shown in Figure 3B. Moreover, 233 commonly downregulated genes and 102 commonly upregulated genes were identified from the immune score/stromal score groups through integrated bioinformatics analysis, and the Venn plot is shown in Figure 3C. These common DEGs are the raw data for subsequent studies.

To investigate the biological functions of the common DEGs, we further performed Gene Ontology (GO) and Kyoto Encyclopedia of Gene and Genomes (KEGG) pathway enrichment analysis. GO enrichment analysis included three subontologies: biological process (BP), cellular component (CC) and molecular function (MF). For BP, the common DEGs mainly enriched in immune response-activating cell surface receptor signaling pathway; For CC, the common DEGs were chiefly enriched in MHC protein complex; For MF, DEGs were principally enriched in immune receptor activity Supplementary Figures 1–3). KEGG pathway enrichment analysis showed that the enrichment of the common DEGs was chiefly concentrated in cell adhesion molecules, systemic lupus erythematosus, and hematopoietic cell lineage (Supplementary Figure 4).

### Identification of a ISCIRGs-based signature

**Figure 4:**
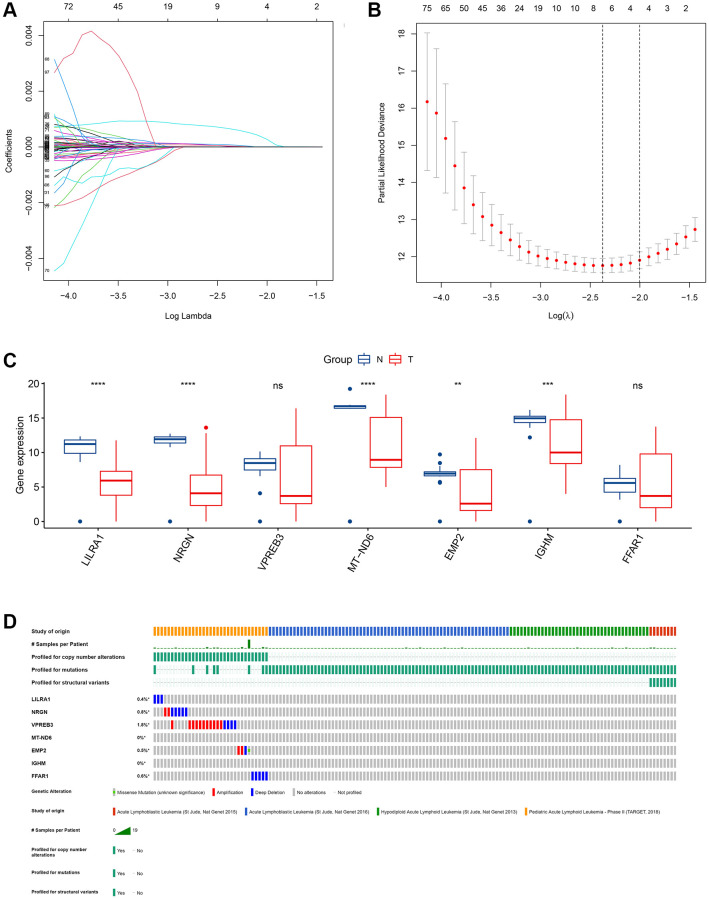
**Identification of an ISCIRG-based signature.** (**A**) The coefficient profile plot was produced against the log(lambda) sequence. A vertical line was drawn at the value selected using ten-fold cross-validation, where an optimal lambda value resulted in ten features with nonzero coefficients. (**B**) Optimal parameter (lambda) selection in the LASSO model used ten-fold cross-validation via minimum criteria. The partial likelihood deviance (binomial deviance) curve was plotted versus the log(lambda) value. Dotted vertical lines were drawn at the optimal values by using the minimum criteria and the I standard error of the minimum criteria. (**C**) The mRNA expression profiles of the seven prognostic ISCIRGs between health and tumor tissues (bulk RNA-seq datasets). ^**^p < 0.01, ^***^p < 0.001 and ^****^p < 0.0001. (**D**) Genetic alterations of seven prognostic ISCIRGs in ALL calculated by cBioPortal database. ^*^p < 0.05.

To investigate the potential value of the common DEGs in predicting the OS of ALL patients, first, KM survival analysis was performed for all common DEGs. The results showed that 335 common DEGs were significantly associated with OS (*p* < 0.05, Supplementary Table 2). Second, univariate Cox analysis was performed, and 317 prognosis-associated ISCIRGs were identified. Least absolute shrinkage and selection operator (LASSO) followed by multivariate Cox analysis identified seven optimal prognosis-related ISCIRGs (i.e., *LILRA1, NRGN, VPREB3, MT-ND6, EMP2, IGHM and FFAR1*) as a risk signature (Figure 4A, 4B, Supplementary Table 3). Based on the multivariate Cox proportional hazards regression model, the expression coefficient of each independent risk gene was obtained, and our prognostic model for predicting prognosis was formed using the following formula: Risk score = Exp_*LILRA1*_ × 0.001077 + Exp_*NRGN*_ × 0.000137 + Exp_*VPREB3*_ × 0.000019 + Exp_*MT-ND6*_ × 0.000005 + Exp_*EMP2*_ × 0.000259 + Exp_*IGHM*_ × 0.000003 + Exp_*FFAR1*_ × 0.000133.

Based on the BM sample of healthy persons and ALL patients from TCGA, we found that, compared to healthy BM, in ALL, the gene expression level of *LILRA1, NRGN, MT-ND6, EMP2,* and *IGHM* were significantly decreased (Figure 4C). Furthermore, based on four datasets of ALL patients (i.e., St Jude, Nat Genet 2013; St Jude, Nat Genet 2015; St Jude, Nat Genet 2016; and TARGET, 2018), the genetic alterations, including amplification and deep deletions, were identified in five genes (i.e., *LILRA1, NRGN, VPREN3, EMP2,* and *FFAR1*), with frequencies ranging from 0.4% to 1.8% (Figure 4D). Representative immunohistochemically pictures of VPREN3 protein expression was shown in Supplementary Figure 5. In addition, the STRING online database and Cytoscape software were used to construct a Protein–Protein Interaction (PPI) network to investigate the interplay among the seven ISCIRGs. The overall network contained 22 nodes and 376 edges (Supplementary Figure 6). Furthermore, GO enrichment analysis showed that the seven ISCIRGs were mainly enriched in mitochondrial electron transport and oxidative phosphorylation (BP), NADH dehydrogenase (ubiquinone) activity (MF), and respirasome (CC). KEGG pathway enrichment analysis demonstrated that these genes were also significantly associated with oxidative phosphorylation (Supplementary Table 4).

### Prognostic value of the ISCIRG-based signature

**Figure 5:**
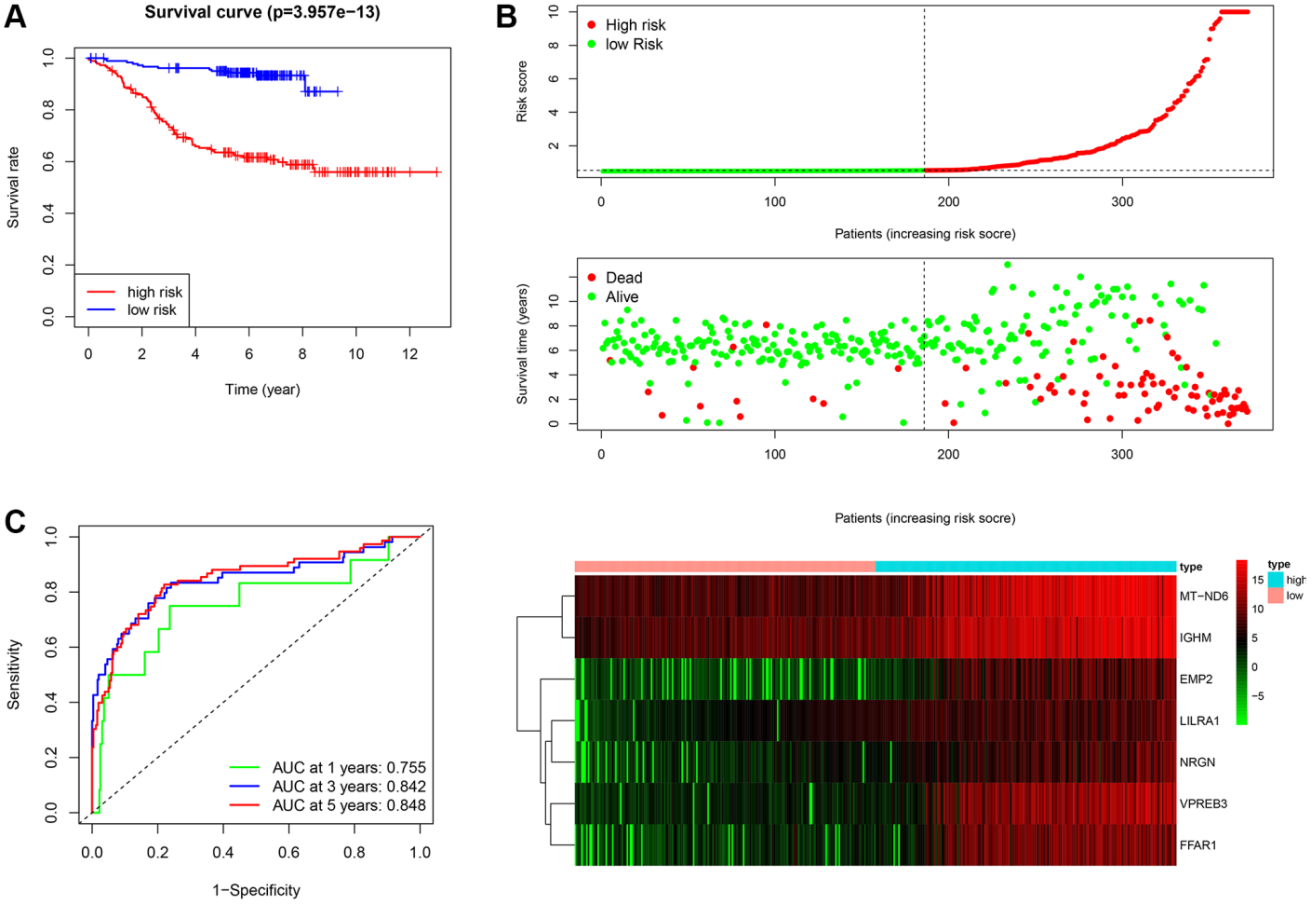
**Assessment of the prognostic value of the ISCIRG signature in training group.** (**A**) KM survival curve for high-risk and low-risk patients. (**B**) Risk score analysis for the high-risk group and low-risk group. Upper panel: Patient survival status and time distributed by the risk score. Middle panel: Risk score curves of the ISCIRG signature. Bottom panel: Heatmaps of the expression levels of the seven ISCIRGs. The colors from green to red indicate the gene expression levels from low to high. (**C**) Time-dependent ROC curve for 1-, 3-, and 5-year OS rates.

**Figure 6:**
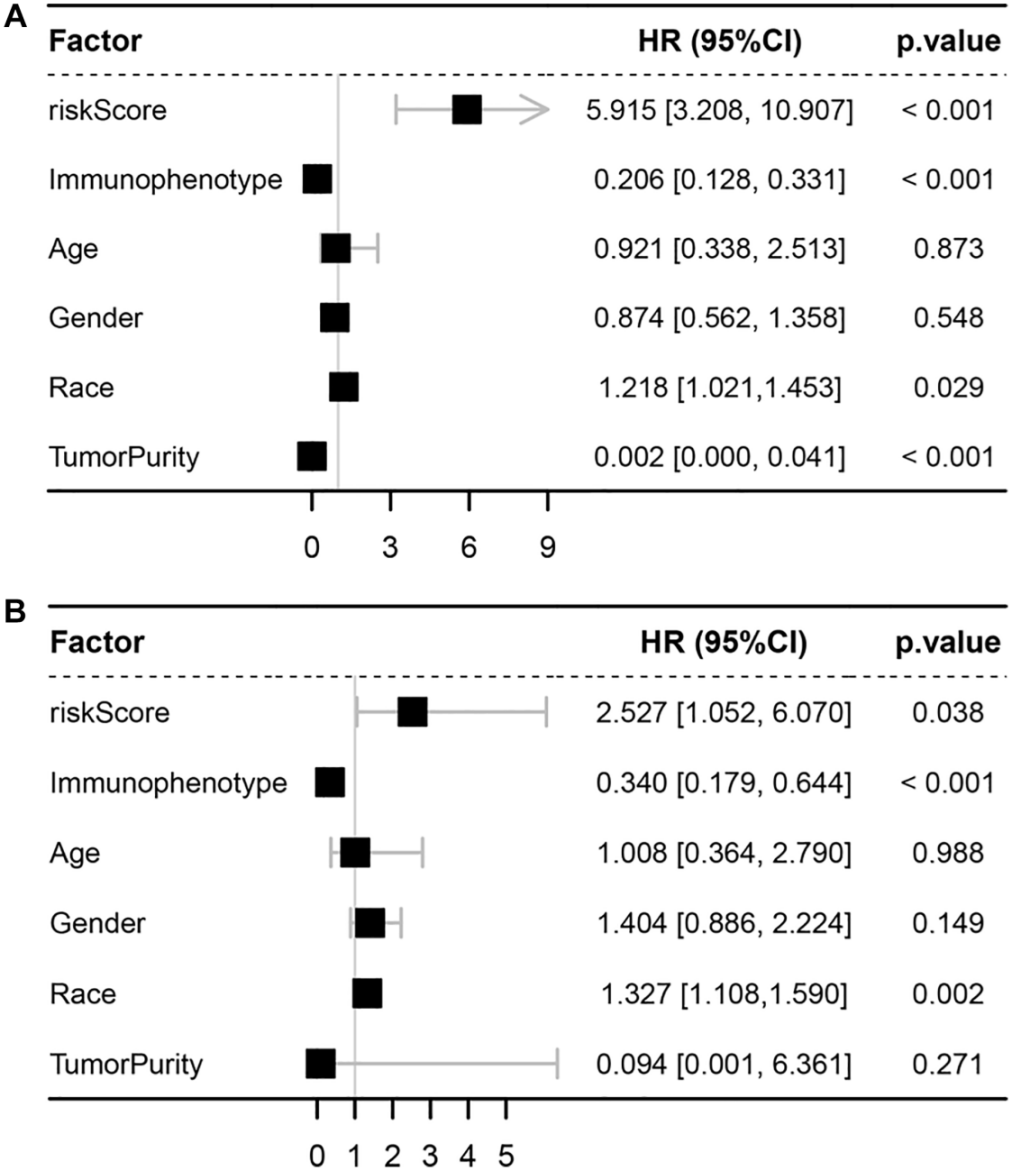
**Univariate and multivariate Cox analyses.** (**A**) Forest plot of univariate Cox analyses. (**B**) Forest plot of multivariate Cox analyses.

After calculating the risk scores of all patients, we used the median risk score as a cutoff value to classify patients of training cohort into high- and low-risk groups. KM survival analysis showed that the patients in high-risk group had significantly lower OS than those in low-risk group (log-rank text *p* < 0.0001, Figure 5A, 5B). Time-dependent ROC analysis confirmed favorable values in predicting OS in this validation set (Figure 5C). We also performed univariate and multivariate Cox regression analysis. Univariate Cox regression analysis confirmed that risk score was a significant prognostic factor (hazard ratio (HR) 95% confidence interval (CI): 5.915 [3.208, 10.907], *p* < 0.001, Figure 6A). Multivariate Cox regression analysis showed that after adjusting for clinicopathological features and tumor purity, the ISCIRG-based signature was still an independent prognostic factor and predictor for ALL patients (HR 95% CI: 2.527 [1.052, 6.070], *p* = 0.038, Figure 6B). In addition, tumor purity was not a factor (*p* = 0.271) influencing the significant association between the ISCIRG-based signature and prognosis in multivariate cox model, demonstrating that this model was not just reporting the level of tumor burden in patients.

**Figure 7:**
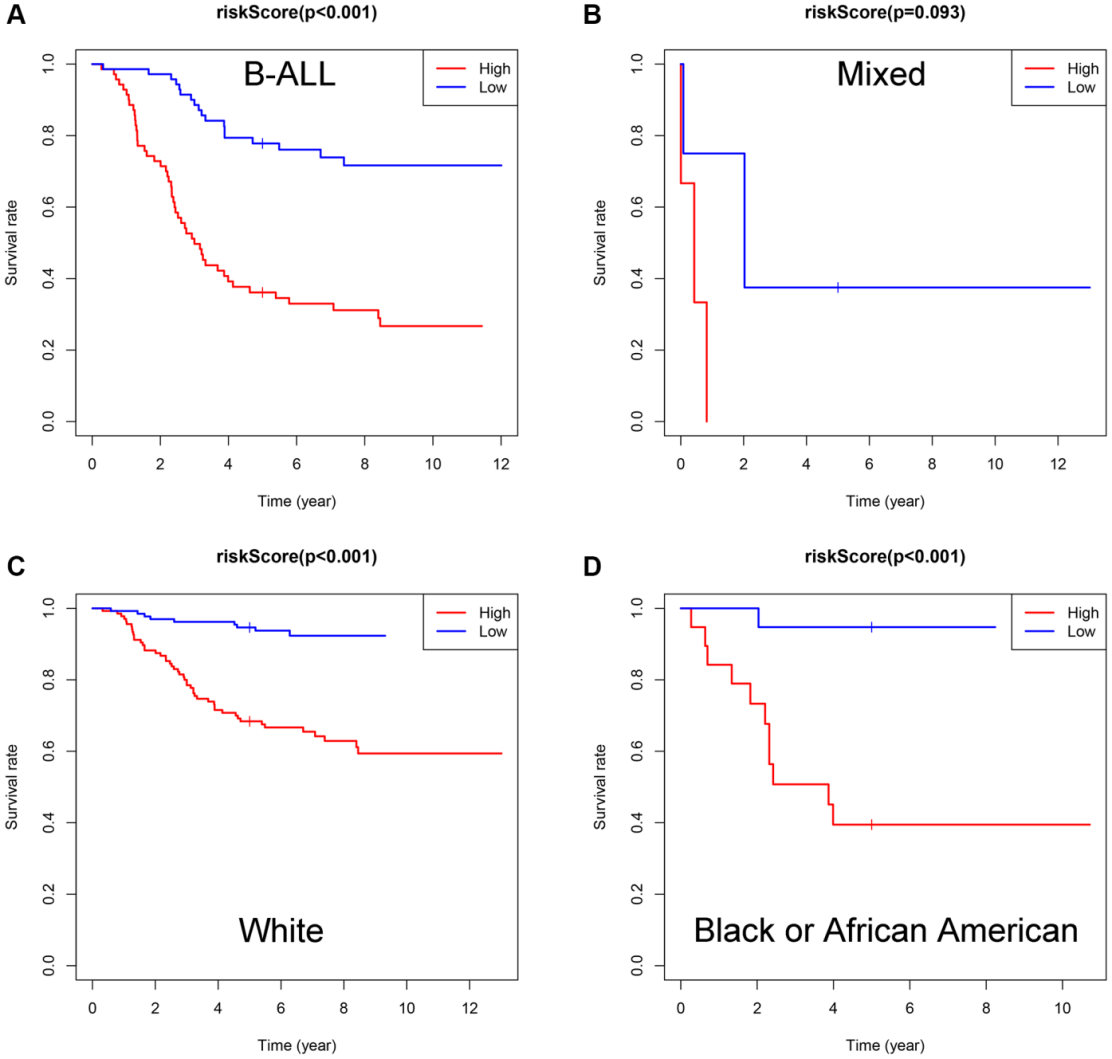
**Evaluation of the *ISCIRGs-based* signature via stratification of patients based on specific clinicopathological features.** (**A**) B-ALL. (**B**) Mixed. (**C**) White. (**D**) Black or African American.

Subsequently, subgroup analysis was performed to further confirm the prognostic value of the gene signature in different clinicopathological factors. The results showed that the association between risk score and OS remained markedly significant after controlling for race and immunophenotype (Figure 7A–7D).

### Association between the ISCIRG-based signature and immune cell infiltration

**Figure 8:**
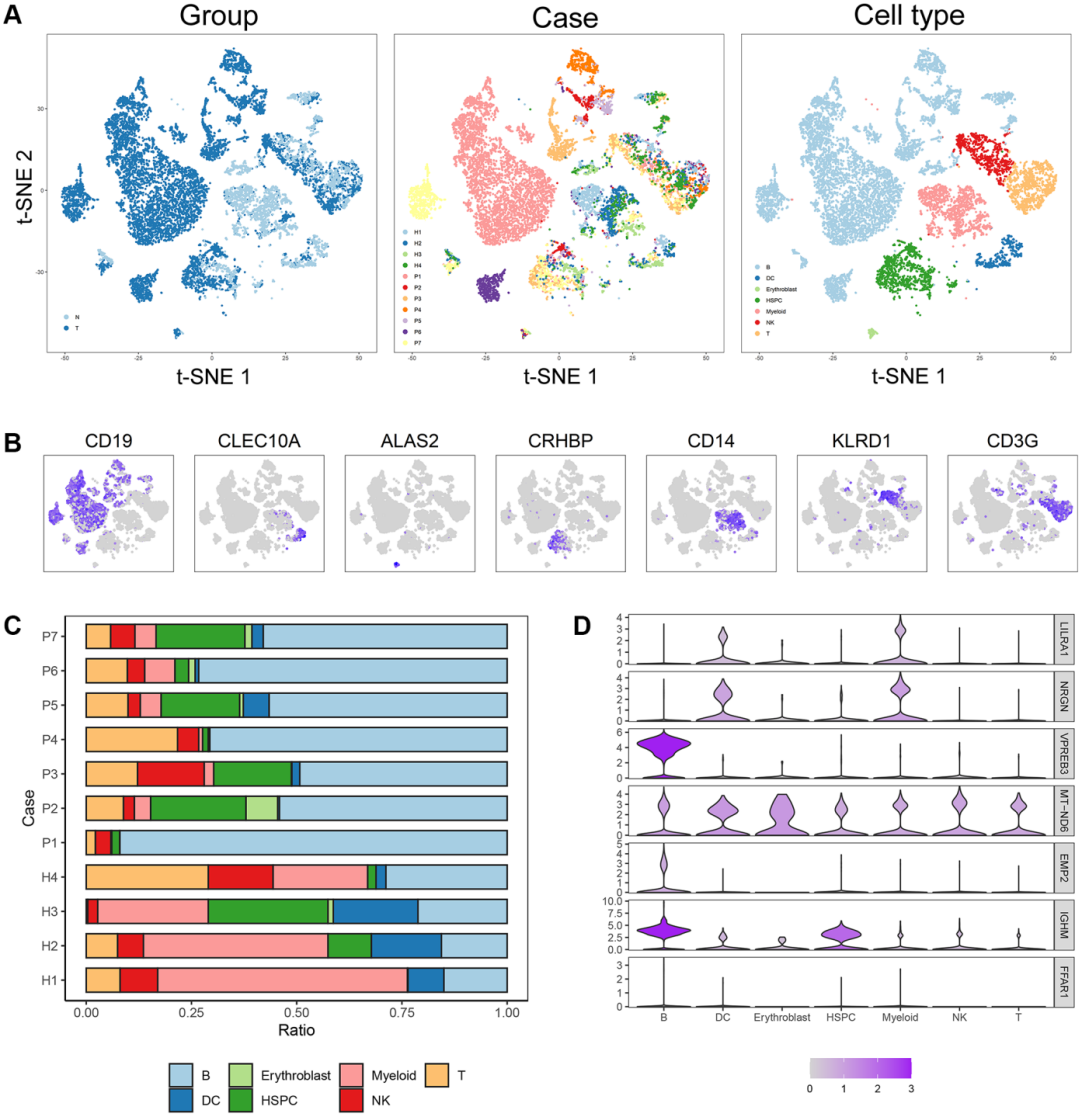
**Cell composition and mRNA expression profiles of seven ISCIRGs in single-cell RNA-seq samples.** (**A**) tSNE of the 27810 cells profiled here, with each cell color-coded for (left to right): its sample group of origin (ALL or health BM), the corresponding case (health cases: H1 to H4, ALL patients: P1 to P7) and the associated cell type. (**B**) Expression of marker genes for the cell types defined above each panel. (**C**) Proportion of cell type in BM of each participant. (**D**) The mRNA expression profiles of the seven ISCIRGs in each type of cell.

Four normal and seven B-ALL single-cell RNA-seq datasets from GSE134759 were included in this analysis. The included samples were collected at the beginning of diagnosis of ALL patients. After quality control, the combined 11 diagnostic BM samples included 27810 cells with a mean and median of 2268 and 2110 detected genes, respectively. The number of genes detected was significantly associated with the sequencing depth. The tSNE algorithm identified 23 separate clusters (Supplementary Figure 7A–7G). Then, 7478 B cells, 933 T cells, 438 dendritic cells (DCs), 77 erythroblast cells, 1068 hematopoietic stem cells and progenitor cells (HSPCs), 1327 myeloid cells and 773 NK cells were identified and annotated based on the marker genes (Figure 8A, 8B). The immune cell infiltration showed a large difference between healthy persons and ALL patients, for some severe patients, such as patient 1, there were almost no other immune cells in BM except tumor cells (i.e., B-cells) (Figure 8C). *LILRA1, NRGN, VPREB3, MT-ND6, EMP2,* and *IGHM* genes were expressed in different degrees in different immune cells and stromal cells, among which *IGHM* and *VPREB3* had significantly higher expression levels in B cells than in other cells, NRGN was expressed in DCs and myeloid cells, and *MT-ND6* was expressed in all kinds of immune cells and myeloid cells of the BM microenvironment (Figure 8D). Moreover, the mRNA expression levels of *LILRA1, VPREB3, EMP2,* and *IGHM* were statistically increased, and the mRNA expression levels of *NRGN* and *MT-ND6* were statistically decreased in ALL sample compared to normal sample (Supplementary Figure 8).

**Figure 9:**
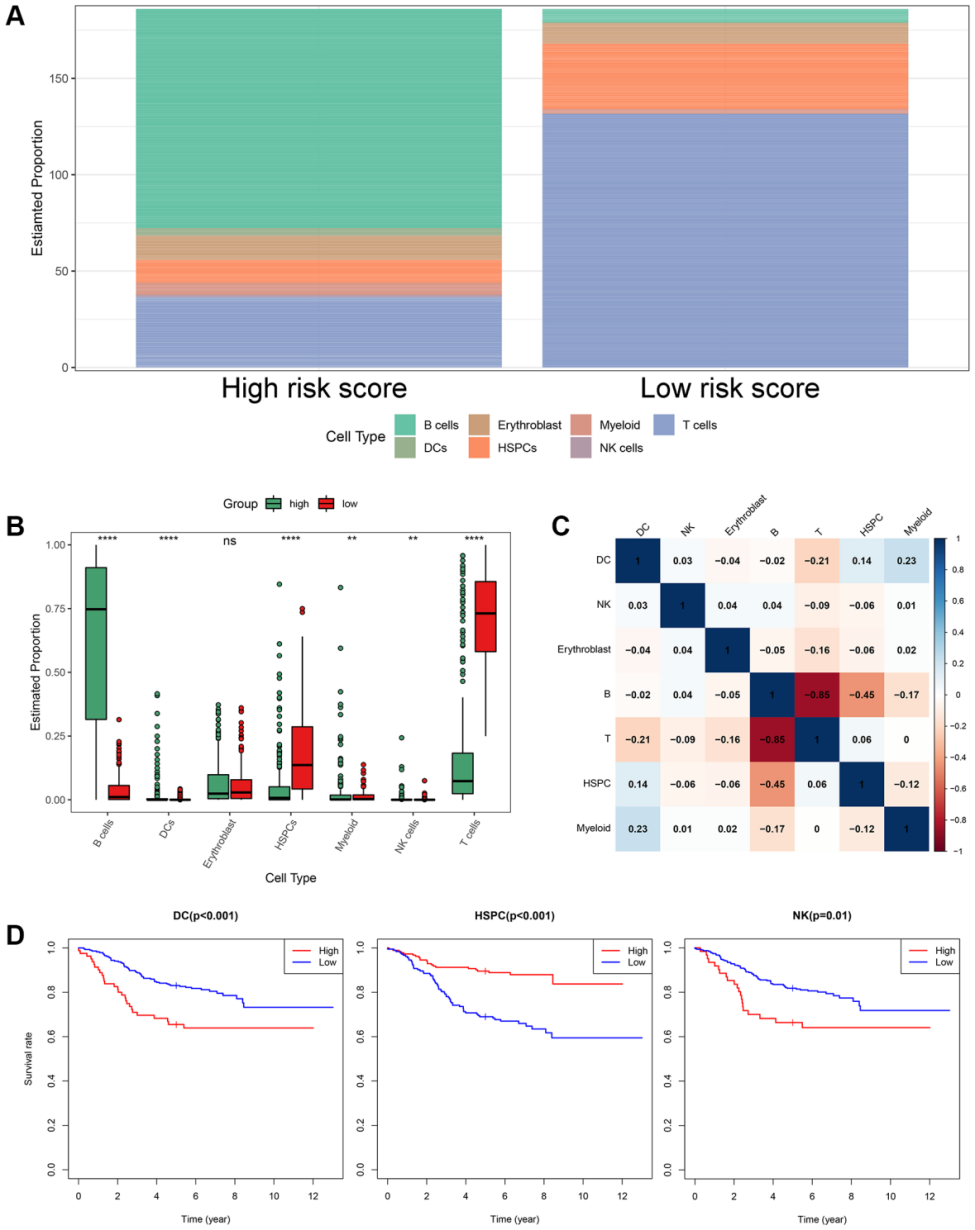
**The landscape of immune cell infiltration between the high- and low-risk groups.** (**A**) Proportion of cell type of the low- and high-risk groups in bulk RNA-seq samples. (**B**) Differential immune cell infiltrates between the high- and low-risk groups. (**C**) Correlation matrix of the relationship between the expression levels of the seven ISCIRGs and differential immune infiltration levels. (**D**) KM survival curves for patients with higher and lower proportions of specific cell.

Based on the annotated single-cell gene expression matrix in the above results, we built a custom signature matrix file by using Cell-type Identification by Estimating Relative Subsets of RNA Transcripts x (CIBERSORTx) (Supplementary Table 5). Subsequently, we used the custom signature matrix file to impute the BM cell fractions for patients of training cohort (Figure 9A). After removing B cells and T cells that may correspond to the immunophenotype of ALL, the results showed that the low-risk group exhibited higher levels of HSPCs. The high-risk group exhibited higher levels of myeloid cells, DCs, and NK cells (Figure 9B). Figure 9C demonstrates correlations between the various immune cells. Furthermore, the survival analysis showed that patients with higher DCs and NK cells had a shorter OS time than those with lower DCs (*p* < 0.001) and NK cells (*p* = 0.01). While, patients with higher HSPCs had a longer OS time than those with lower HSPCs (*p* < 0.001, Figure 9D).

### Construction and validation of clinically applicable prognostic nomogram

**Figure 10:**
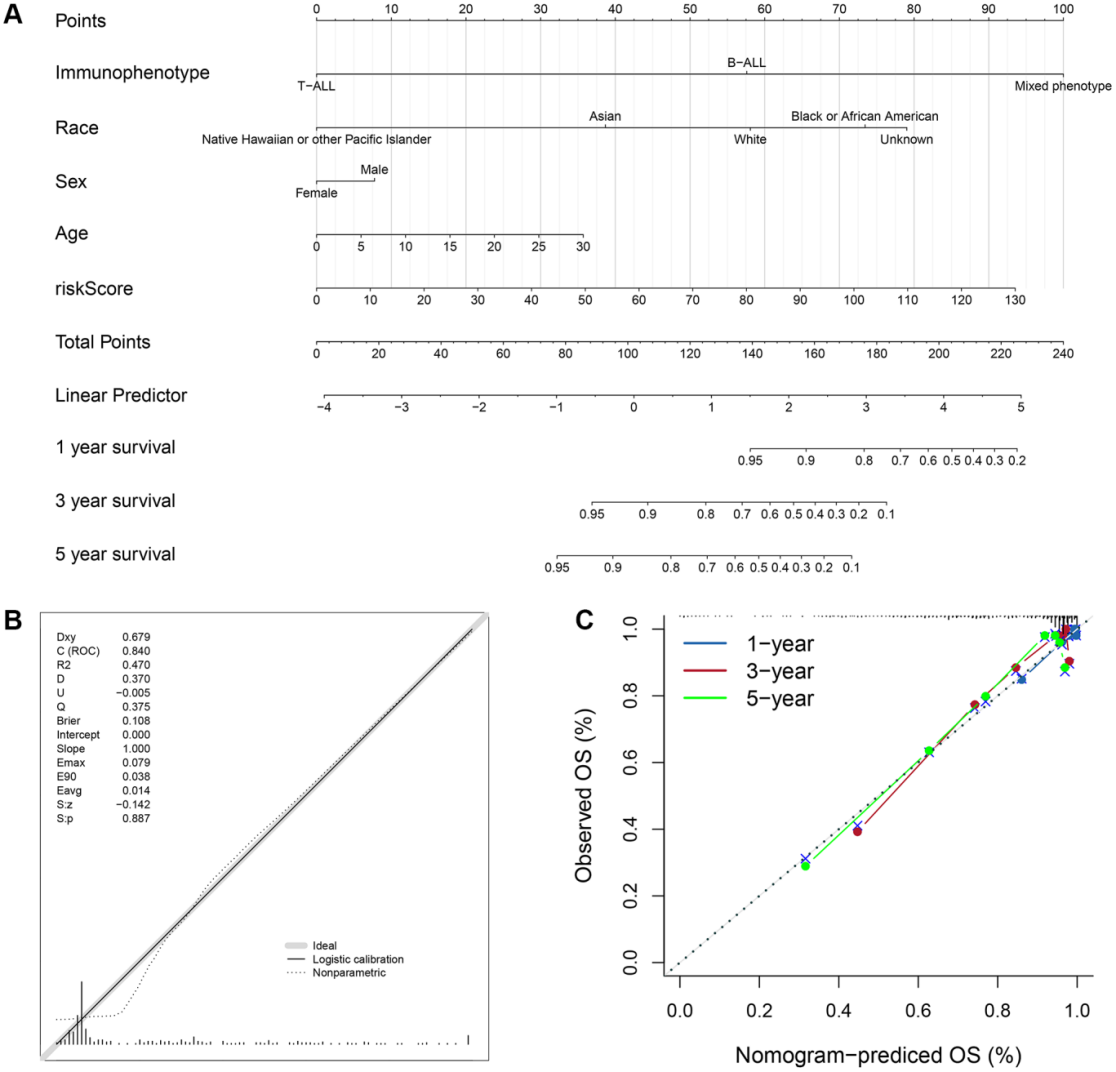
**Prognostic nomogram to predict the 1-, 3-, and 5-year OS of ALL patients.** (**A**) Nomogram model to predict the prognosis of ALL patients. (**B**) Calibration test for the prognostic nomogram. (**C**) Calibration plot of the prognostic nomogram for predicting OS at 1-, 3-, and 5-years.

Based on the four valuable factors (immunophenotype, race, age and gender) and the ISCIRG-based signature in the bulk RNA-seq data set of training group, a prognostic nomogram was developed to provide a clinically applicable quantitative approach for individual OS prediction (Figure 10A). The calibration test indicated the perfect prediction ability of this model (concordance index (C-index): 0.802 95% CI: 0.7473−0.8572, S: *p* = 0.887, ROC = 0.840, Figure 10B). The calibration plots suggested that the agreement between the predicted 1-, 3-, and 5-year OS rates and actual observations was excellent (Figure 10C). These results suggest that the prognostic nomogram is reliable and can be applied for ALL patients.

To validate the feasibility and robustness of our nomogram, we performed a similar analysis process for dataset of validation group. KM survival analysis showed that the population with higher risk score also had significantly lower OS than those with lower risk score (log-rank text *p* = 0.03, Supplementary Figure 9A, 9C). Time-dependent ROC analysis confirmed favorable values in predicting OS in this validation set (Supplementary Figure 9B). And the calibration test showed the good prediction ability as well (C-index: 0.6861 95% CI: 0.5777−0.7946, S: *p* = 0.962, ROC = 0.778, Supplementary Figure 10A). The calibration plots also suggested that the agreement between the predicted 1-, 3-, and 5-year OS rates and actual observations was good (Supplementary Figure 10B). Besides, we further validated the prognostic significance of this model by analyzing two external validation datasets, including GSE13576 (*n* = 197) and GSE50999 (*n* = 43). The results showed that patients (GSE13576) with pediatric B-ALL in higher risk score group had higher early and late relapse rates (19.4%) compared to those in lower risk score group (13.3%, Supplementary Figure 11, Supplementary Table 6), and patients (GSE50999) with T-ALL in higher risk score group also had a higher relapse rate (28.6%) than those in lower risk score group (13.6%, Supplementary Figure 12, Supplementary Table 6).

**Figure 11:**
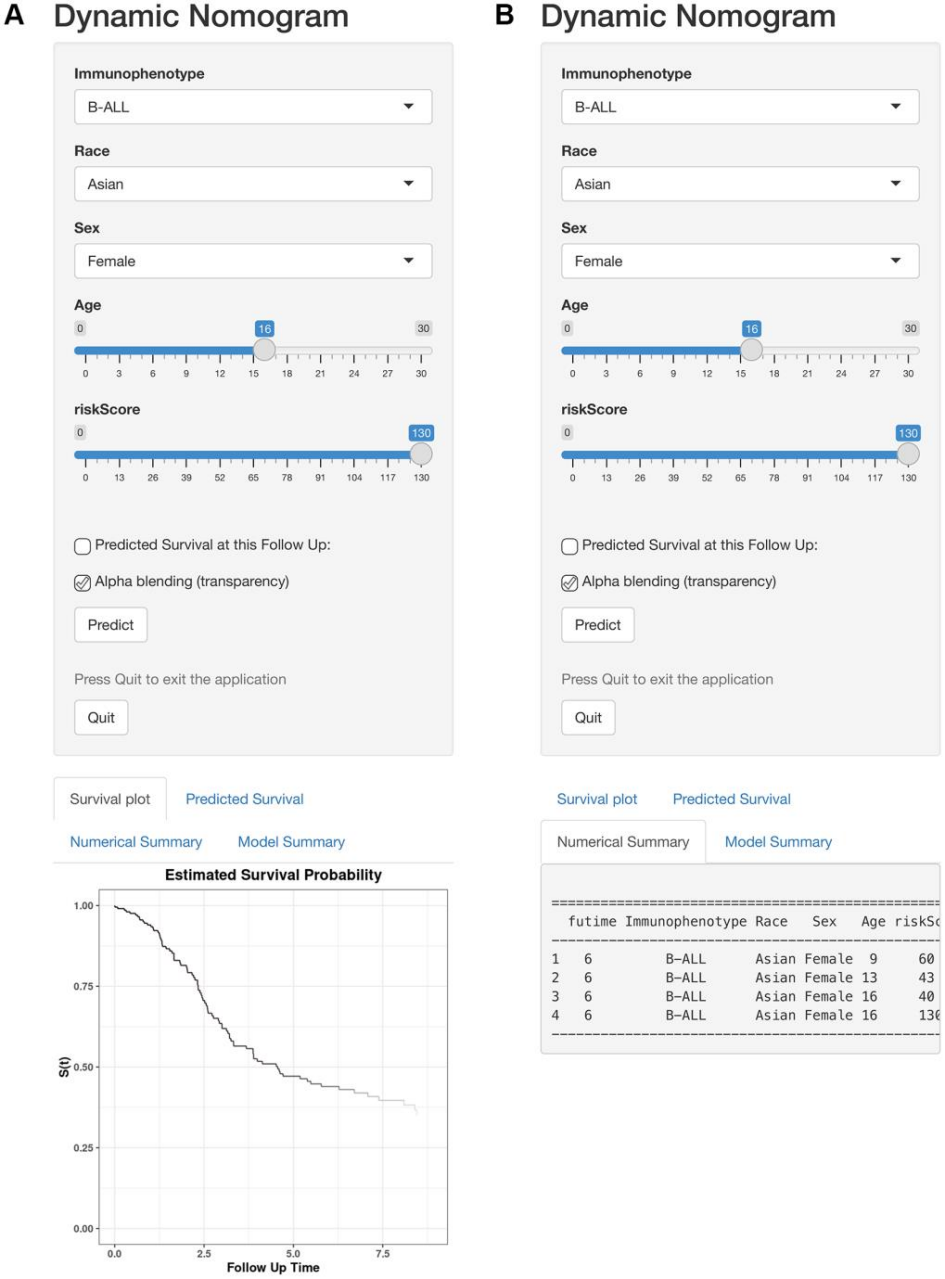
**Web-based calculator for predicting OS in patients with ALL.** (**A**) Web-based overall survival rate calculator. (**B**) The 95% CI of the web-based progression-free survival rate.

Furthermore, to visualize and facilitate the clinical use of the prognostic nomogram, we developed an easy-to- operate web-based model that predicted the OS of ALL based on the established nomogram (Figure 11A, 11B). The estimated probability of disease progression could be obtained by drawing a vertical line from the total point axis to the outcome axis.

## DISCUSSION

ALL is a rapidly progressive hematologic malignancy whose maintenance and progression are highly dependent upon the interaction between immune/stromal cells and the nonmalignant microenvironment [24]. Therefore, in the early stage of disease progression, adequate assessments of the infiltration of immune/stromal cells and accurate prediction tools are essential for treatment decision-making and prognostic evaluation in patients with ALL. This study investigated immune/stromal cell infiltration and assessed the involvement of ISCIRGs in prognosis in patients with ALL. First, the scores regarding immune/stromal cell infiltration were proved to be of prognostic value. Second, a ISCIRGs-based signature was constructed. Third, seven key ISCIRGs were confirmed that had prognostic value and were associated with the infiltration of specific types of immune cells based on the analysis results combined single-cell RNA-seq and bulk RNA-seq datasets. Finally, a prognostic nomogram composed of the ISCIRGs-based signature, age, gender, race and immunophenotype was successfully developed and validated for predicting the prognosis of ALL patients.

Some previous studies have showed that there are differences in the prognosis of ALL patients with various immunophenotypes. For example, for T-ALL, 50% of adult patients have poor prognosis after chemotherapy [25], while, for B-ALL, some patients still have more than 60% relapse rate even after treatment with CAR T therapy [26]. Since three immunophenotypes (i.e., T-ALL, B-ALL and MPAL) were included in this study, we first calculated the immune/stromal scores for patient groups with three immunophenotypes, respectively, and found that the scores were significantly different between three patient groups. This suggested that the immune/stromal cells of patients with various immunophenotypes were in different states, and this difference might be a nonnegligible factor leading to poor prognosis of various immunophenotypes. Subsequently, we found that patients with different race also had different stromal scores. It is known that racial and ethnic disparities persist in the outcome and prognosis of ALL [27]. Therefore, the proportion and states of stromal cells might be two important factors leading to the different prognosis of various races. Then, the KM curve showed that the immune and stromal scores were significantly associated with OS, respectively. Thus, both the immune and stromal cell infiltration were meaningful in predicting OS, and it is necessary to identify prognostic genes based on immune scores, stromal scores or both.

After obtaining the common DEGs from the low vs. high immune score/stromal score groups, we performed a functional enrichment analysis for these genes, and found that these genes mainly enriched in immune system. This result not only verified the reliability of the ESTIMATE algorithm, but also indicated that ISCIRGs mainly regulated the immune system. Previous studies have found that some individual genes regulated immune system are crucial factors for ALL with poor prognosis, such as IKZF1 [28]. While, in this study, the prognostic signature was constructed based on seven ISCIRGs (i.e., *LILRA1, NRGN, VPREB3, MT-ND6, EMP2, IGHM* and *FFAR1*). The OS association of this prognostic signature (*p* = 3.957e−13) was significantly higher than ESTIMATE immune scores-based signature (*p* = 0.015) or stromal scores-based signature (*p* = 0.003), suggesting the great potential of prognostic prediction.

Among the seven ISCIRGs, *VPREB3* and *IGHM* have been confirmed that are associated with the prognosis of ALL [29], and *MT-ND6* are found mutation in ALL patients [30]. In terms of each gene, the higher expression of all seven ISCIRGs was positively associated with shorter OS time. The major function of the seven genes included: *LILRA1* is to regulate immune responses by interacting with MHC class I ligands [31]; *NRGN* is a postsynaptic protein kinase substrate that binds calmodulin in the absence of calcium [32]; *VPREB3* and *IGHM* is thought to be involved in B cell maturation [33], mutation or absence can cause either an arrest or a severe impairment at the pro-B cell stage [34, 35]; *MT-ND6* involved in mitochondrial electron transport, NADH to ubiquinone and mitochondrial respiratory chain complex I assembly [36]; *EMP2* regulates cell membrane composition, and up-regulation of this gene has been linked to cancer progression in multiple different tissues [37, 38]; and *FFAR1* involved in the metabolic regulation of insulin secretion [39]. In terms of the synergies of these genes, the results of PPI network analysis and functional enrichment analysis for the seven DEGs showed that these genes were mainly enriched in mitochondrial electron transport and oxidative phosphorylation. Jaramillo et al. [40] found that Rho (0) malignant T cells with impaired mitochondrial electron transport chain function had lower sensitivity to the combination treatment than wild-type cells, and Chen et al. [41] confirmed that oxidative phosphorylation can enhance resistance to chemotherapy in B-ALL. And all patients included in the survival analysis were treated with conventional combined chemotherapy. Therefore, the high feasibility of the prognostic signature may also be related to the reduction of combined chemotherapy sensitivity caused by the differential expression of the seven genes. These genes may also be the key hints for overcoming resistance to immunotherapy.

Subsequently, we investigated the expression of the seven genes in various cell types by analyzing the single-cell RNA-datasets, and further performed the difference in the proportion of immune cells in high- and low-risk groups by integrating single-cell and bulk RNA-seq datasets. In the single-cell RNA-seq datasets, we found that due to the accumulation of a large number of immature lymphocytes, the immune cells in the BM of ALL patients were significantly reduced compared to the healthy BM, and the types of other immune cells (except for immature lymphocytes) showed differences in each patients. And this difference was related to the ISCIRGs-based signature and the prognosis of patients of bulk RNA-seq datasets. Previous studies have suggested that some immune cells could predict the prognosis of all patients, for example, Hohtari et al. [10] and Zhou et al. [11] found that the proportion of PD1+TIM3+ double-positive CD4+ T cells could differentiate the poor survival group of B-ALL, and Duault et al. [12] revealed that increased frequencies of activated cytokine-producing NK cells could independently predict poor clinical outcome in ALL patient. Our results showed that the patients in high-risk group had significant higher proportions of DCs and NK cells compared to those in low-risk group, and higher proportions of DCs and NK cells had significant shorter OS time compared to lower proportions. The results regarding association between NK cells and poor prognosis were consistent with previous study [12], while the results of DCs were contrary to previous cognition. DCs were thought to be able to present tumor antigens, stimulating immune system to play an anti-tumor role in ALL [42]. However, our results showed that higher proportions of DCs was no benefit for good prognosis for the first time. Given that the seven ISCIRGs-based signature are directly related to the proportion and even the status of immune cells, future studies are required to elucidate the mechanisms associated with prognosis of these genes in specific immune cells (e.g., DCs).

Nomogram is a multivariable regression model that widely used in studies to predict clinical outcomes with intuitive visual presentations [43]. In this study, we constructed a prognostic nomogram based on the ISCIRGs-based signature and clinicopathological factors of training group to predict ALL patient outcome. The results of validation group and long-term follow-up examinations indicated the high reliability of this nomogram. To our knowledge, this nomogram is the first to predict ALL patient survival based on ISCIRGs. And compared with the prediction models published in previous studies [21–23], our prediction model has higher accuracy and wider application range. In addition, considering the imbalance of the prognostic model, we included two more independent studies related to the prognosis of ALL patients (external validation cohorts) to further validate the prognostic value of the ISCIRGs-based signature. The results showed that the higher risk groups of the both external validation cohorts had higher relapse rates compared to the lower risk groups. A large number of clinical data have confirmed that relapse is the major reason for poor prognosis of ALL patients, once relapse, the completed cure rate will be less than 40% [44, 45]. And the major factor leading to relapse is the resistance of patients to combined chemotherapy. Therefore, the results of two external validation cohorts not only further verified the great prognostic prediction ability of the ISCIRGs-based signature, but also further suggested the potential correlation between seven prognostic genes and resistance of combined chemotherapy. Furthermore, we established the corresponding web-based calculator. The scoring system and web-based calculator may help clinicians make survival predictions based on clinicopathological factors and cellular differentiation information of the BM microenvironment and further suggest better treatment options.

This study still has some limitations. First, the results of this study were obtained only through an integrated bioinformatics analysis. Although we verified the results through one validation cohort and two external validation cohorts, and included some immunohistochemical pictures of crucial ISCIRGs in both health and ALL BM, this is still inadequate. Before the results are used in clinical prediction, clinical experimental validation is needed to confirm them, and the underlying mechanisms associated with the prognostic significance of the identified ISCIRGs in ALL need to be further investigated. Second, although we have tried our best to search the RNA-seq datasets of ALL patients with prognostic information through various databases, literatures and search engines, the datasets containing survival information are still limited. Third, due to the lack of clinicopathologic information in the included datasets, other potential risk factors influenced prognosis (e.g., chemotherapy, molecular drugs, SNP and CNV) were not considered.

In summary, we highlight the immune/stromal cell infiltration differences in the BM environment and their essential roles in predicting the clinical outcome of ALL patients, constructing a prognostic model based on the ISCIRG signature and clinicopathologic factors. This model could serve as a reliable tool for predicting outcomes and determining treatment strategies in patients with ALL.

## MATERIALS AND METHODS

### Raw data retrieval and calculation of immune/stromal cell infiltration

The gene expression file datasets of training group as well as corresponding metadata and clinical profiles were obtained from TCGA database after restricting “Disease Type” to “lymphoid leukemia”, “Experimental Strategy” to “RNA-Seq”, “Data Category” to “transcriptome profiling” and “Data Type” to “Gene Expression Quantification”. The format of the downloaded data is “HTSeq-Counts”. All patients included in this study suffered from ALL. We excluded the following datasets: 1) not BM sample; 2) without follow-up time; 3) unknown death or not; and 4) without detail clinical characteristics. The gene expression file dataset and clinical profile of validation group were selected from TARGET project database. We merged the data of ALL patients in Phases I, II and III, and then excluded the following datasets: 1) duplicate patients with training cohort; 2) not BM sample; 3) without follow-up time; 4) unknown death or not; and 5) without detail clinical characteristic. The calculation of immune score, stromal score and tumor purity by employing the ESTIMATE algorithm for all downloaded dataset was performed by a custom script of Python 3.9.5 (Python Software Foundation, Delaware, USA) and “estimate” R package of R software 4.0.5 (R Foundation for Statistical Computing, Vienna, Austria) [46]. The stromal and immune scores were calculated by perform single-sample gene set-enrichment analysis (ssGSEA) [47, 48], and the tumor purity was calculated by using the following formula:

Tumor purity=cos(0.6049872018 + 0.0001467884 ×ESTIMATE score)

ESTIMATE score represents the sum of stromal score and immune score [15].

### Common DEGs identification

Based on the immune/stromal score, the included patients were classified into high- and low-score groups. The DEGs of training group were analyzed and identified through the “limma” R package, and the cutoff values were set as | fold change (FC)| > 2 and *p* < 0.05 [49]. The heatmap and volcano plots were generated by the “ggplot2” and “pheatmap” R packages, respectively [50]. The identification of the common DEGs from the immune score and stromal score groups was processed by Python script [46].

### Construction and validation of the prognostic genes signature

First, KM survival analysis was used to illuminate correlations between immune/stromal scores and OS as well as each common DEG and OS, and the log-rank test was utilized to assess the statistical significance of the correlation [51]. Second, univariate Cox regression analysis was performed to evaluate the association between the expression levels of common DEGs and OS. Third, the identified prognosis-related genes (*p* < 0.05) were screened by LASSO and multivariate Cox regression analyses [52]. An ISCIRG-based signature was constructed by using the following formula:

$$Riskscore=\sum_{\text{i}=1}^{\text{N}}\text{(ExpGENEi}\times \text{β}\text{i)}$$

ExpGENEi represents the expression level of the identified ISCIRGs, and βi represents the regression coefficient calculated by multivariate Cox analysis [53].

Fourth, based on the risk score model, patients were stratified into either the low score (low-risk) group or the high score (high-risk) group. KM survival analysis was used to estimate the OS of these two groups. The predictive accuracy of this model was assessed by Harrell’s C-index and time-dependent ROC curve analysis within 1-, 3- and 5-years [54]. Univariate and multivariate Cox regression analyses were performed to assess whether the predictive performance of this model could be independent of tumor purity or other clinicopathologic factors.

### Construction of prognostic nomogram and validation of model

Based on the results of univariate and multivariate Cox regression analyses, the identified independent prognostic factors were used to develop a prognostic nomogram for predicting the 1-, 3-, and 5-year survival outcomes by the “rms” R package. Subsequently, the C-index, *U* test, ROC and calibration plot were used to assess the discrimination performance of this prognostic nomogram in training and validation cohorts [54]. Furthermore, two external validation datasets (i.e., GSE13576 and GSE28703) from Gene Expression Omnibus (GEO) database were used to verify the significance of the prognostic model. The risk scores were first calculated. Then, we verified the clinical significance of our model by comparing the prognostic information of patients. Moreover, to facilitate clinical application, we constructed a visualization tool with a web-based calculator [55].

### Single-cell RNA-seq data processing

Single-cell RNA-seq datasets of ALL (GSE134759) were downloaded from GEO database. First, we merged patient and healthy sample datasets by the “Seurat” R package [56]. Then, we removed the low-quality cells based on the following standards: 1) genes detected in fewer than three cells; 2) cells with fewer than fifty total detected genes; 3) cells with more than 10% mitochondrion-expressed genes; and 4) cells with fewer than 1500 or more than 4000 expressed genes. The 2000 most variable genes were generated and used to perform principal component analysis (PCA). The t-distributed stochastic neighbor embedding (t-SNE) algorithm was used to compute 20 initial PCs and perform cluster classification analysis. Subsequently, we used the Wilcoxon rank sum test with Bonferroni multiple-comparison correction to determine cluster marker genes, and *p* < 0.05 and | log2(FC) | > 0.25 were set as cutoffs. Finally, each cell cluster was annotated by the “singleR” R package and then verified with broad cell type markers (Supplementary Table 7) [57, 58].

### Immune cell infiltration analysis in high and low risk populations

We calculated the fractions of immune cell subsets in ALL samples (bulk RNA-seq) with the CIBERSORTx algorithm. First, based on the annotated single-cell gene expression matrix in the above results (single-cell RNA-seq), a custom signature matrix file was constructed by using CIBERSORTx Create Signature Matrix module. Then, the proportion of each types of cell in bulk RNA-seq datasets were calculated by using CIBERSORTx Impute Cell Fractions module [59]. Correlation relation matrix was performed by the “corrgram” and “corrplot” R packages. KM survival analysis was used to assess correlations between the proportion of each types of cell and OS [60].

### Functional analysis

GO and KEGG pathway enrichment analysis as well as the interrelation analysis were calculated and plotted by using the ClueGO plug-in in Cytoscape software 3.6.1 (National Resource for Network Biology, California, USA), and *p* < 0.05 was set as the cutoff [61]. The cBio Cancer Genomics Portal (cBioPortal) database was used to evaluate the mutations and copy number variations in tumor patients [60]. STRING database was used to assess the gene-encoded proteins and PPI information [62]. The downloaded PPI information file was subsequently established as a PPI network using Cytoscape software. The modular analysis for the PPI network was performed by the MCODE plug-in in Cytoscape based on score and node number, and the most significant module was identified [63].

The complete method design of this study is shown in Supplementary Figure 13.

### Data availability statement

Publicly available datasets were analyzed in this study. These data can be found as follow links: The datasets of training cohort from TCGA, https://tcga-data.nci.nih.gov/tcga/ (accessed on 4 May 2021); The datasets of validation cohort from TARGET project database, https://target-data.nci.nih.gov/Public/ALL/mRNA-seq/ (accessed on 10 April 2022); Single-cell RNA-seq datasets from GEO database, https://www.ncbi.nlm.nih.gov/geo/query/acc.cgi?acc=GSE134759 (accessed on 1 April 2022); The datasets of external validation datasets, https://www.ncbi.nlm.nih.gov/geo/query/acc.cgi?acc=GSE13576, https://www.ncbi.nlm.nih.gov/geo/query/acc.cgi (accessed on 10 August 2022); cBioPortal database, http://www.cbioportal.org/ (accessed on 22 January 2022); STRING database, https://cn.string-db.org/ (accessed on 22 January 2022); CIBERSORTx, https://cibersortx.stanford.edu/ (accessed on 10 August 2022); The web-based calculator that we constructed in this study could be found and used in link: https://yuwenliang6666.shinyapps.io/DynNomapp/. All custom Python and R script of this study could be made available through collaboration.

## Supplementary Materials

Supplementary Figures

Supplementary Table 1

Supplementary Table 2

Supplementary Tables 3-4 and 7

Supplementary Table 5

Supplementary Table 6

## References

[1] Dander E, Palmi C, D'Amico G, Cazzaniga G. The Bone Marrow Niche in B-Cell Acute Lymphoblastic Leukemia: The Role of Microenvironment from Pre-Leukemia to Overt Leukemia. Int J Mol Sci. 2021; 22:4426. 10.3390/ijms22094426 33922612 PMC8122951

[2] Alexander TB, Orgel E. Mixed Phenotype Acute Leukemia: Current Approaches to Diagnosis and Treatment. Curr Oncol Rep. 2021; 23:22. 10.1007/s11912-020-01010-w 33544265

[3] Yu WL, Hua ZC. Chimeric Antigen Receptor T-cell (CAR T) Therapy for Hematologic and Solid Malignancies: Efficacy and Safety-A Systematic Review with Meta-Analysis. Cancers (Basel). 2019; 11:47. 10.3390/cancers11010047 30621018 PMC6356949

[4] Bassan R, Hoelzer D. Modern therapy of acute lymphoblastic leukemia. J Clin Oncol. 2011; 29:532–43. 10.1200/JCO.2010.30.1382 21220592

[5] Hu J, Chen Z, Bao L, Zhou L, Hou Y, Liu L, Xiong M, Zhang Y, Wang B, Tao Z, Chen K. Single-Cell Transcriptome Analysis Reveals Intratumoral Heterogeneity in ccRCC, which Results in Different Clinical Outcomes. Mol Ther. 2020; 28:1658–72. 10.1016/j.ymthe.2020.04.023 32396851 PMC7335756

[6] McConkey DJ, Choi W. Molecular Subtypes of Bladder Cancer. Curr Oncol Rep. 2018; 20:77. 10.1007/s11912-018-0727-5 30128829

[7] Sarode P, Schaefer MB, Grimminger F, Seeger W, Savai R. Macrophage and Tumor Cell Cross-Talk Is Fundamental for Lung Tumor Progression: We Need to Talk. Front Oncol. 2020; 10:324. 10.3389/fonc.2020.00324 32219066 PMC7078651

[8] Zuo S, Wei M, Wang S, Dong J, Wei J. Pan-Cancer Analysis of Immune Cell Infiltration Identifies a Prognostic Immune-Cell Characteristic Score (ICCS) in Lung Adenocarcinoma. Front Immunol. 2020; 11:1218. 10.3389/fimmu.2020.01218 32714316 PMC7344231

[9] Burt R, Dey A, Aref S, Aguiar M, Akarca A, Bailey K, Day W, Hooper S, Kirkwood A, Kirschner K, Lee SW, Lo Celso C, Manji J, et al. Activated stromal cells transfer mitochondria to rescue acute lymphoblastic leukemia cells from oxidative stress. Blood. 2019; 134:1415–29. 10.1182/blood.2019001398 31501154 PMC6856969

[10] Hohtari H, Brück O, Blom S, Turkki R, Sinisalo M, Kovanen PE, Kallioniemi O, Pellinen T, Porkka K, Mustjoki S. Immune cell constitution in bone marrow microenvironment predicts outcome in adult ALL. Leukemia. 2019; 33:1570–82. 10.1038/s41375-018-0360-1 30635636 PMC6755974

[11] Zhou Q, Munger ME, Veenstra RG, Weigel BJ, Hirashima M, Munn DH, Murphy WJ, Azuma M, Anderson AC, Kuchroo VK, Blazar BR. Coexpression of Tim-3 and PD-1 identifies a CD8+ T-cell exhaustion phenotype in mice with disseminated acute myelogenous leukemia. Blood. 2011; 117:4501–10. 10.1182/blood-2010-10-310425 21385853 PMC3099570

[12] Duault C, Kumar A, Taghi Khani A, Lee SJ, Yang L, Huang M, Hurtz C, Manning B, Ghoda L, McDonald T, Lacayo NJ, Sakamoto KM, Carroll M, et al. Activated natural killer cells predict poor clinical prognosis in high-risk B- and T-cell acute lymphoblastic leukemia. Blood. 2021; 138:1465–80. 10.1182/blood.2020009871 34077953 PMC8532198

[13] Jurisic V. Multiomic analysis of cytokines in immuno-oncology. Expert Rev Proteomics. 2020; 17:663–74. 10.1080/14789450.2020.1845654 33131355

[14] Konjević GM, Vuletić AM, Mirjačić Martinović KM, Larsen AK, Jurišić VB. The role of cytokines in the regulation of NK cells in the tumor environment. Cytokine. 2019; 117:30–40. 10.1016/j.cyto.2019.02.001 30784898

[15] Yoshihara K, Shahmoradgoli M, Martínez E, Vegesna R, Kim H, Torres-Garcia W, Treviño V, Shen H, Laird PW, Levine DA, Carter SL, Getz G, Stemke-Hale K, et al. Inferring tumour purity and stromal and immune cell admixture from expression data. Nat Commun. 2013; 4:2612. 10.1038/ncomms3612 24113773 PMC3826632

[16] Ni J, Liu S, Qi F, Li X, Yu S, Feng J, Zheng Y. Screening TCGA database for prognostic genes in lower grade glioma microenvironment. Ann Transl Med. 2020; 8:209. 10.21037/atm.2020.01.73 32309356 PMC7154476

[17] Shi J, Jiang D, Yang S, Zhang X, Wang J, Liu Y, Sun Y, Lu Y, Yang K. LPAR1, Correlated With Immune Infiltrates, Is a Potential Prognostic Biomarker in Prostate Cancer. Front Oncol. 2020; 10:846. 10.3389/fonc.2020.00846 32656075 PMC7325998

[18] Pan JH, Zhou H, Cooper L, Huang JL, Zhu SB, Zhao XX, Ding H, Pan YL, Rong L. LAYN Is a Prognostic Biomarker and Correlated With Immune Infiltrates in Gastric and Colon Cancers. Front Immunol. 2019; 10:6. 10.3389/fimmu.2019.00006 30761122 PMC6362421

[19] Li X, Wen D, Li X, Yao C, Chong W, Chen H. Identification of an Immune Signature Predicting Prognosis Risk and Lymphocyte Infiltration in Colon Cancer. Front Immunol. 2020; 11:1678. 10.3389/fimmu.2020.01678 33013820 PMC7497441

[20] Yan H, Qu J, Cao W, Liu Y, Zheng G, Zhang E, Cai Z. Identification of prognostic genes in the acute myeloid leukemia immune microenvironment based on TCGA data analysis. Cancer Immunol Immunother. 2019; 68:1971–8. 10.1007/s00262-019-02408-7 31650199 PMC11028253

[21] Chen Z, Yang F, Liu H, Fan F, Lin Y, Zhou J, Cai Y, Zhang X, Wu Y, Mao R, Zhang T. Identification of a nomogram based on an 8-lncRNA signature as a novel diagnostic biomarker for childhood acute lymphoblastic leukemia. Aging (Albany NY). 2021; 13:15548–68. 10.18632/aging.203116 34106877 PMC8221355

[22] Mao R, Hu S, Zhang Y, Du F, Zhang Y, Liu Y, Zhang T. Prognostic Nomogram for Childhood Acute Lymphoblastic Leukemia: A Comprehensive Analysis of 673 Patients. Front Oncol. 2020; 10:1673. 10.3389/fonc.2020.01673 33014835 PMC7511595

[23] Zhang D, Cheng Y, Fan J, Yao J, Zhao Z, Jiang Y, Li Y, Zuo Z, Tang Y, Guo Y. A Nomogram for the Prediction of Progression and Overall Survival in Childhood Acute Lymphoblastic Leukemia. Front Oncol. 2020; 10:1550. 10.3389/fonc.2020.01550 32984014 PMC7477348

[24] Passaro D, Quang CT, Ghysdael J. Microenvironmental cues for T-cell acute lymphoblastic leukemia development. Immunol Rev. 2016; 271:156–72. 10.1111/imr.12402 27088913

[25] Chen Y, Cheng Y, Suo P, Yan C, Wang Y, Chen Y, Han W, Xu L, Zhang X, Liu K, Chang L, Xiao L, Huang X. Donor-derived CD19-targeted T cell infusion induces minimal residual disease-negative remission in relapsed B-cell acute lymphoblastic leukaemia with no response to donor lymphocyte infusions after haploidentical haematopoietic stem cell transplantation. Br J Haematol. 2017; 179:598–605. 10.1111/bjh.14923 29076142

[26] Qian M, Zhao X, Devidas M, Yang W, Gocho Y, Smith C, Gastier-Foster JM, Li Y, Xu H, Zhang S, Jeha S, Zhai X, Sanda T, et al. Genome-Wide Association Study of Susceptibility Loci for T-Cell Acute Lymphoblastic Leukemia in Children. J Natl Cancer Inst. 2019; 111:1350–7. 10.1093/jnci/djz043 30938820 PMC6910193

[27] Lee SHR, Antillon-Klussmann F, Pei D, Yang W, Roberts KG, Li Z, Devidas M, Yang W, Najera C, Lin HP, Tan AM, Ariffin H, Cheng C, et al. Association of Genetic Ancestry With the Molecular Subtypes and Prognosis of Childhood Acute Lymphoblastic Leukemia. JAMA Oncol. 2022; 8:354–63. 10.1001/jamaoncol.2021.6826 35084434 PMC8796058

[28] Payne KJ, Dovat S. Ikaros and tumor suppression in acute lymphoblastic leukemia. Crit Rev Oncog. 2011; 16:3–12. 10.1615/critrevoncog.v16.i1-2.20 22150303 PMC3243972

[29] Chen D, Zheng J, Gerasimcik N, Lagerstedt K, Sjögren H, Abrahamsson J, Fogelstrand L, Mårtensson IL. The Expression Pattern of the Pre-B Cell Receptor Components Correlates with Cellular Stage and Clinical Outcome in Acute Lymphoblastic Leukemia. PLoS One. 2016; 11:e0162638. 10.1371/journal.pone.0162638 27611867 PMC5017602

[30] Järviaho T, Hurme-Niiranen A, Soini HK, Niinimäki R, Möttönen M, Savolainen ER, Hinttala R, Harila-Saari A, Uusimaa J. Novel non-neutral mitochondrial DNA mutations found in childhood acute lymphoblastic leukemia. Clin Genet. 2018; 93:275–85. 10.1111/cge.13100 28708239

[31] Pilsbury LE, Allen RL, Vordermeier M. Modulation of Toll-like receptor activity by leukocyte Ig-like receptors and their effects during bacterial infection. Mediators Inflamm. 2010; 2010:536478. 10.1155/2010/536478 20634939 PMC2903975

[32] Nakajima R, Hattori S, Funasaka T, Huang FL, Miyakawa T. Decreased nesting behavior, selective increases in locomotor activity in a novel environment, and paradoxically increased open arm exploration in Neurogranin knockout mice. Neuropsychopharmacol Rep. 2021; 41:111–6. 10.1002/npr2.12150 33270377 PMC8182962

[33] Felizola SJ, Katsu K, Ise K, Nakamura Y, Arai Y, Satoh F, Sasano H. Pre-B Lymphocyte Protein 3 (VPREB3) Expression in the Adrenal Cortex: Precedent for non-Immunological Roles in Normal and Neoplastic Human Tissues. Endocr Pathol. 2015; 26:119–28. 10.1007/s12022-015-9366-7 25861052

[34] Conley ME, Burrows PD. Plugging the leaky pre-B cell receptor. J Immunol. 2010; 184:1127–9. 10.4049/jimmunol.0990113 20089707

[35] Mårtensson IL, Almqvist N, Grimsholm O, Bernardi AI. The pre-B cell receptor checkpoint. FEBS Lett. 2010; 584:2572–9. 10.1016/j.febslet.2010.04.057 20420836

[36] Cao K, Lv W, Wang X, Dong S, Liu X, Yang T, Xu J, Zeng M, Zou X, Zhao D, Ma Q, Lin M, Long J, et al. Hypermethylation of Hepatic Mitochondrial *ND6* Provokes Systemic Insulin Resistance. Adv Sci (Weinh). 2021; 8:2004507. 10.1002/advs.202004507 34141522 PMC8188198

[37] Dillard C, Kiyohara M, Mah V, McDermott SP, Bazzoun D, Tsui J, Chan AM, Haddad G, Pellegrini M, Chang YL, Elshimali Y, Wu Y, Vadgama JV, et al. EMP2 Is a Novel Regulator of Stemness in Breast Cancer Cells. Mol Cancer Ther. 2020; 19:1682–95. 10.1158/1535-7163.MCT-19-0850 32451329 PMC7415657

[38] Patel KS, Kejriwal S, Sun MM, Thammachantha S, Duong C, Chan A, Cherian N, Romiyo P, Gordon LK, Yong W, Wadehra M, Yang I. Identification of epithelial membrane protein 2 (EMP2) as a molecular marker and correlate for angiogenesis in meningioma. J Neurooncol. 2020; 147:15–24. 10.1007/s11060-020-03401-2 31981014 PMC7080560

[39] Guzmán-Silva A, Martínez-Morales JC, Medina LDC, Romero-Ávila MT, Villegas-Comonfort S, Solís KH, García-Sáinz JA. Mutation of putative phosphorylation sites in the free fatty acid receptor 1: Effects on signaling, receptor phosphorylation, and internalization. Mol Cell Endocrinol. 2022; 545:111573. 10.1016/j.mce.2022.111573 35065200

[40] Jaramillo MC, Briehl MM, Batinic-Haberle I, Tome ME. Manganese (III) meso-tetrakis N-ethylpyridinium-2-yl porphyrin acts as a pro-oxidant to inhibit electron transport chain proteins, modulate bioenergetics, and enhance the response to chemotherapy in lymphoma cells. Free Radic Biol Med. 2015; 83:89–100. 10.1016/j.freeradbiomed.2015.01.031 25725417 PMC4441837

[41] Chen C, Hao X, Lai X, Liu L, Zhu J, Shao H, Huang D, Gu H, Zhang T, Yu Z, Xie L, Zhang X, Yang Y, et al. Oxidative phosphorylation enhances the leukemogenic capacity and resistance to chemotherapy of B cell acute lymphoblastic leukemia. Sci Adv. 2021; 7:eabd6280. 10.1126/sciadv.abd6280 33692103 PMC7946372

[42] Weber G, Caruana I, Rouce RH, Barrett AJ, Gerdemann U, Leen AM, Rabin KR, Bollard CM. Generation of tumor antigen-specific T cell lines from pediatric patients with acute lymphoblastic leukemia--implications for immunotherapy. Clin Cancer Res. 2013; 19:5079–91. 10.1158/1078-0432.CCR-13-0955 23838315 PMC3778051

[43] Furnari FB, Cloughesy TF, Cavenee WK, Mischel PS. Heterogeneity of epidermal growth factor receptor signalling networks in glioblastoma. Nat Rev Cancer. 2015; 15:302–10. 10.1038/nrc3918 25855404 PMC4875778

[44] Pui CH, Carroll WL, Meshinchi S, Arceci RJ. Biology, risk stratification, and therapy of pediatric acute leukemias: an update. J Clin Oncol. 2011; 29:551–65. 10.1200/JCO.2010.30.7405 21220611 PMC3071256

[45] Parker C, Waters R, Leighton C, Hancock J, Sutton R, Moorman AV, Ancliff P, Morgan M, Masurekar A, Goulden N, Green N, Révész T, Darbyshire P, et al. Effect of mitoxantrone on outcome of children with first relapse of acute lymphoblastic leukaemia (ALL R3): an open-label randomised trial. Lancet. 2010; 376:2009–17. 10.1016/S0140-6736(10)62002-8 21131038 PMC3010035

[46] Ye Z, Bing A, Zhao S, Yi S, Zhan X. Comprehensive analysis of spliceosome genes and their mutants across 27 cancer types in 9070 patients: clinically relevant outcomes in the context of 3P medicine. EPMA J. 2022; 13:335–50. 10.1007/s13167-022-00279-0 35719132 PMC9203615

[47] Barbie DA, Tamayo P, Boehm JS, Kim SY, Moody SE, Dunn IF, Schinzel AC, Sandy P, Meylan E, Scholl C, Fröhling S, Chan EM, Sos ML, et al. Systematic RNA interference reveals that oncogenic KRAS-driven cancers require TBK1. Nature. 2009; 462:108–12. 10.1038/nature08460 19847166 PMC2783335

[48] Verhaak RG, Hoadley KA, Purdom E, Wang V, Qi Y, Wilkerson MD, Miller CR, Ding L, Golub T, Mesirov JP, Alexe G, Lawrence M, O'Kelly M, et al, and Cancer Genome Atlas Research Network. Integrated genomic analysis identifies clinically relevant subtypes of glioblastoma characterized by abnormalities in PDGFRA, IDH1, EGFR, and NF1. Cancer Cell. 2010; 17:98–110. 10.1016/j.ccr.2009.12.020 20129251 PMC2818769

[49] Ritchie ME, Phipson B, Wu D, Hu Y, Law CW, Shi W, Smyth GK. limma powers differential expression analyses for RNA-sequencing and microarray studies. Nucleic Acids Res. 2015; 43:e47. 10.1093/nar/gkv007 25605792 PMC4402510

[50] Li T, Zhang C, Zhao G, Zhang X, Hao M, Hassan S, Zhang M, Zheng H, Yang D, Liu L, Mehraein-Ghomi F, Bai X, Chen K, et al. Data analysis of PD-1 antibody in the treatment of melanoma patients. Data Brief. 2020; 30:105523. 10.1016/j.dib.2020.105523 32322636 PMC7168734

[51] Bland JM, Altman DG. Survival probabilities (the Kaplan-Meier method). BMJ. 1998; 317:1572. 10.1136/bmj.317.7172.1572 9836663 PMC1114388

[52] Nagashima K, Sato Y. Information criteria for Firth's penalized partial likelihood approach in Cox regression models. Stat Med. 2017; 36:3422–36. 10.1002/sim.7368 28608396 PMC6084330

[53] Wang Z, Gao L, Guo X, Feng C, Lian W, Deng K, Xing B. Development and validation of a nomogram with an autophagy-related gene signature for predicting survival in patients with glioblastoma. Aging (Albany NY). 2019; 11:12246–69. 10.18632/aging.102566 31844032 PMC6949068

[54] Alba AC, Agoritsas T, Walsh M, Hanna S, Iorio A, Devereaux PJ, McGinn T, Guyatt G. Discrimination and Calibration of Clinical Prediction Models: Users' Guides to the Medical Literature. JAMA. 2017; 318:1377–84. 10.1001/jama.2017.12126 29049590

[55] Tang Y, Wang J, Chen G, Ye W, Yan N, Feng Z. A simple-to-use web-based calculator for survival prediction in Parkinson's disease. Aging (Albany NY). 2021; 13:5238–49. 10.18632/aging.202443 33535176 PMC7950310

[56] Macosko EZ, Basu A, Satija R, Nemesh J, Shekhar K, Goldman M, Tirosh I, Bialas AR, Kamitaki N, Martersteck EM, Trombetta JJ, Weitz DA, Sanes JR, et al. Highly Parallel Genome-wide Expression Profiling of Individual Cells Using Nanoliter Droplets. Cell. 2015; 161:1202–14. 10.1016/j.cell.2015.05.002 26000488 PMC4481139

[57] Wang Z, Guo X, Gao L, Wang Y, Ma W, Xing B. Glioblastoma cell differentiation trajectory predicts the immunotherapy response and overall survival of patients. Aging (Albany NY). 2020; 12:18297–321. 10.18632/aging.103695 32957084 PMC7585071

[58] Witkowski MT, Dolgalev I, Evensen NA, Ma C, Chambers T, Roberts KG, Sreeram S, Dai Y, Tikhonova AN, Lasry A, Qu C, Pei D, Cheng C, et al. Extensive Remodeling of the Immune Microenvironment in B Cell Acute Lymphoblastic Leukemia. Cancer Cell. 2020; 37:867–82.e12. 10.1016/j.ccell.2020.04.015 32470390 PMC7341535

[59] Newman AM, Steen CB, Liu CL, Gentles AJ, Chaudhuri AA, Scherer F, Khodadoust MS, Esfahani MS, Luca BA, Steiner D, Diehn M, Alizadeh AA. Determining cell type abundance and expression from bulk tissues with digital cytometry. Nat Biotechnol. 2019; 37:773–82. 10.1038/s41587-019-0114-2 31061481 PMC6610714

[60] Xu Q, Chen Y. An Aging-Related Gene Signature-Based Model for Risk Stratification and Prognosis Prediction in Lung Adenocarcinoma. Front Cell Dev Biol. 2021; 9:685379. 10.3389/fcell.2021.685379 34277626 PMC8283194

[61] Bindea G, Mlecnik B, Hackl H, Charoentong P, Tosolini M, Kirilovsky A, Fridman WH, Pagès F, Trajanoski Z, Galon J. ClueGO: a Cytoscape plug-in to decipher functionally grouped gene ontology and pathway annotation networks. Bioinformatics. 2009; 25:1091–3. 10.1093/bioinformatics/btp101 19237447 PMC2666812

[62] Cook HV, Doncheva NT, Szklarczyk D, von Mering C, Jensen LJ. Viruses.STRING: A Virus-Host Protein-Protein Interaction Database. Viruses. 2018; 10:519. 10.3390/v10100519 30249048 PMC6213343

[63] Shen S, Kong J, Qiu Y, Yang X, Wang W, Yan L. Identification of core genes and outcomes in hepatocellular carcinoma by bioinformatics analysis. J Cell Biochem. 2019; 120:10069–81. 10.1002/jcb.28290 30525236

