# Supplementary material for: Identification of immune and stromal cell infiltration-related gene signature for prognosis prediction in acute lymphoblastic leukemia
Source: Aging (Albany NY). 2022 Sep 19;14(18):7470–504. doi: 10.18632/aging.204292 (PMC9550239; doi:10.18632/aging.204292)
Supplement: Supplementary Figures [file aging-14-204292-s001.pdf]

SUPPLEMENTARY FIGURES

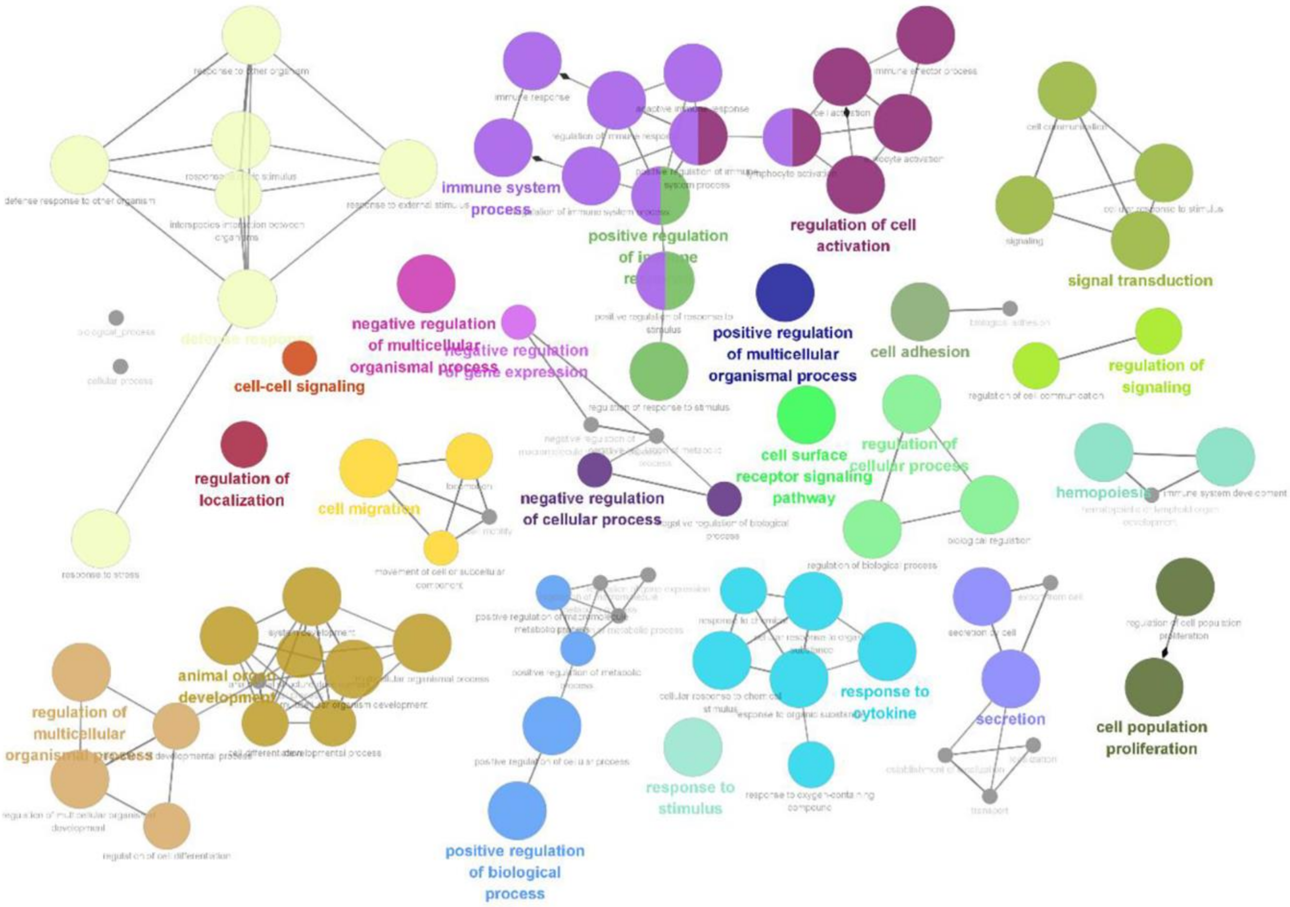

Supplementary Figure 1. Interrelation analysis was performed by assessing the biological process of the common DEGs.

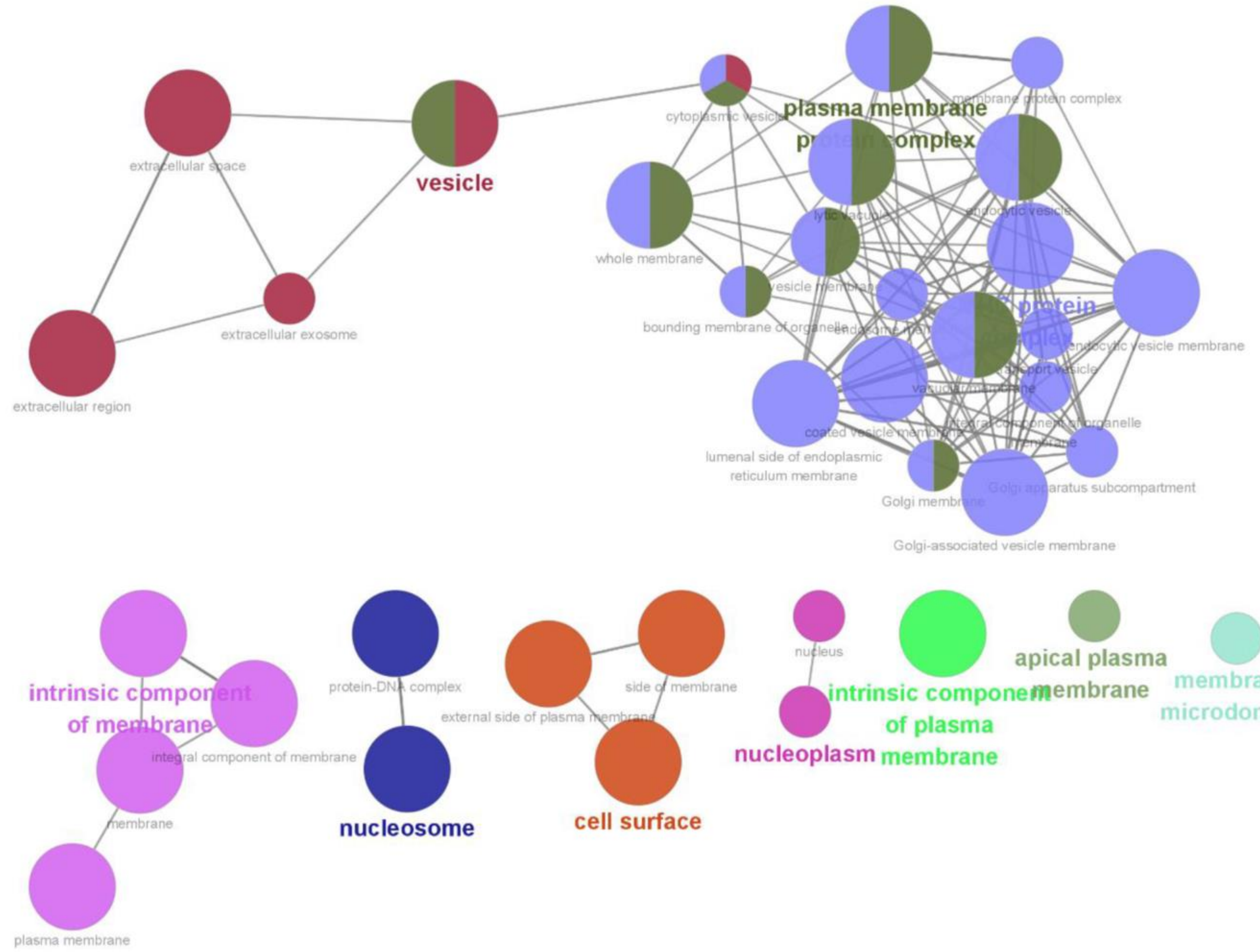

Supplementary Figure 2. Interrelation analysis was performed by assessing the cellular components of the common DEGs.

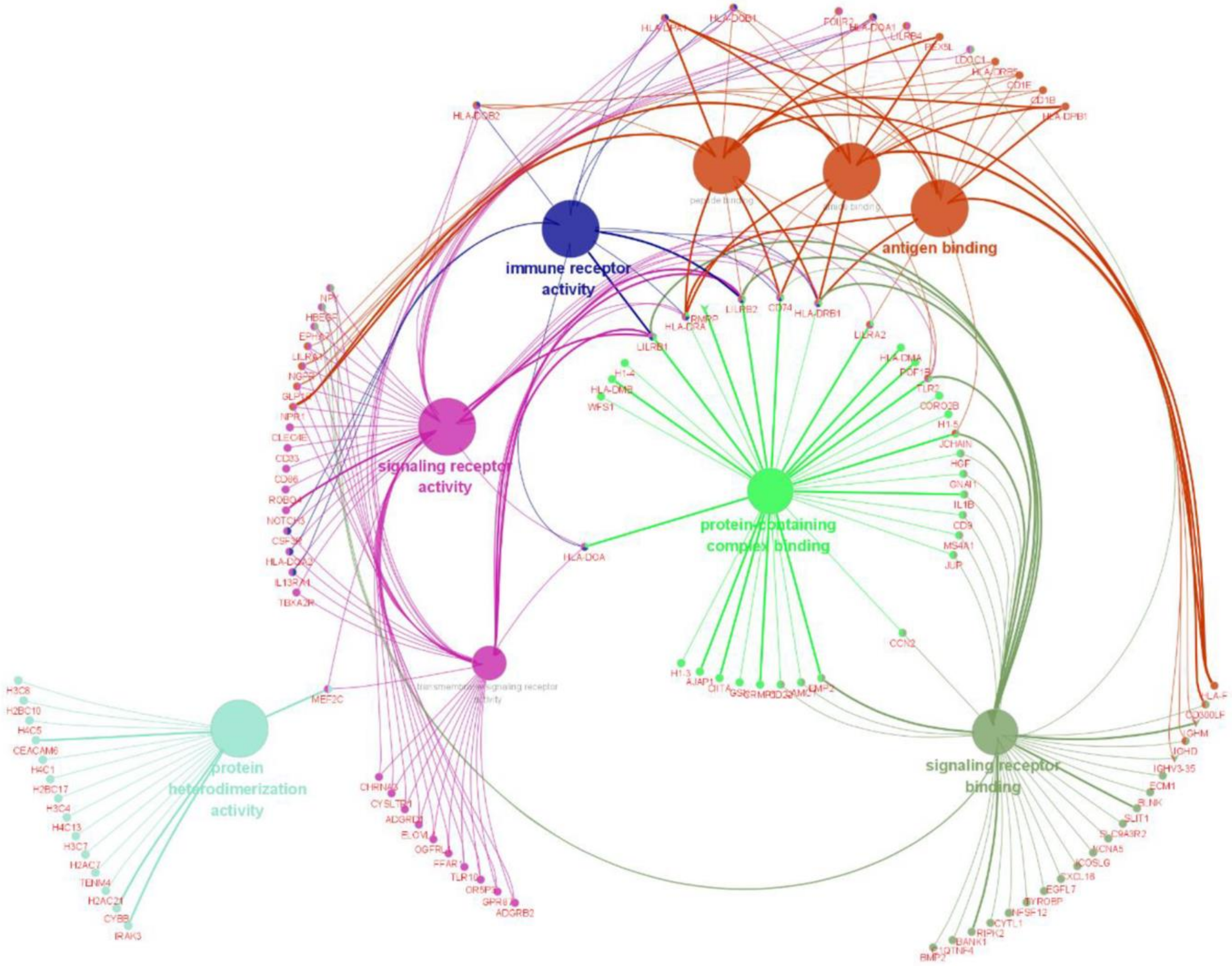

Supplementary Figure 3. Interrelation analysis was performed by assessing the molecular functions of the common DEGs.

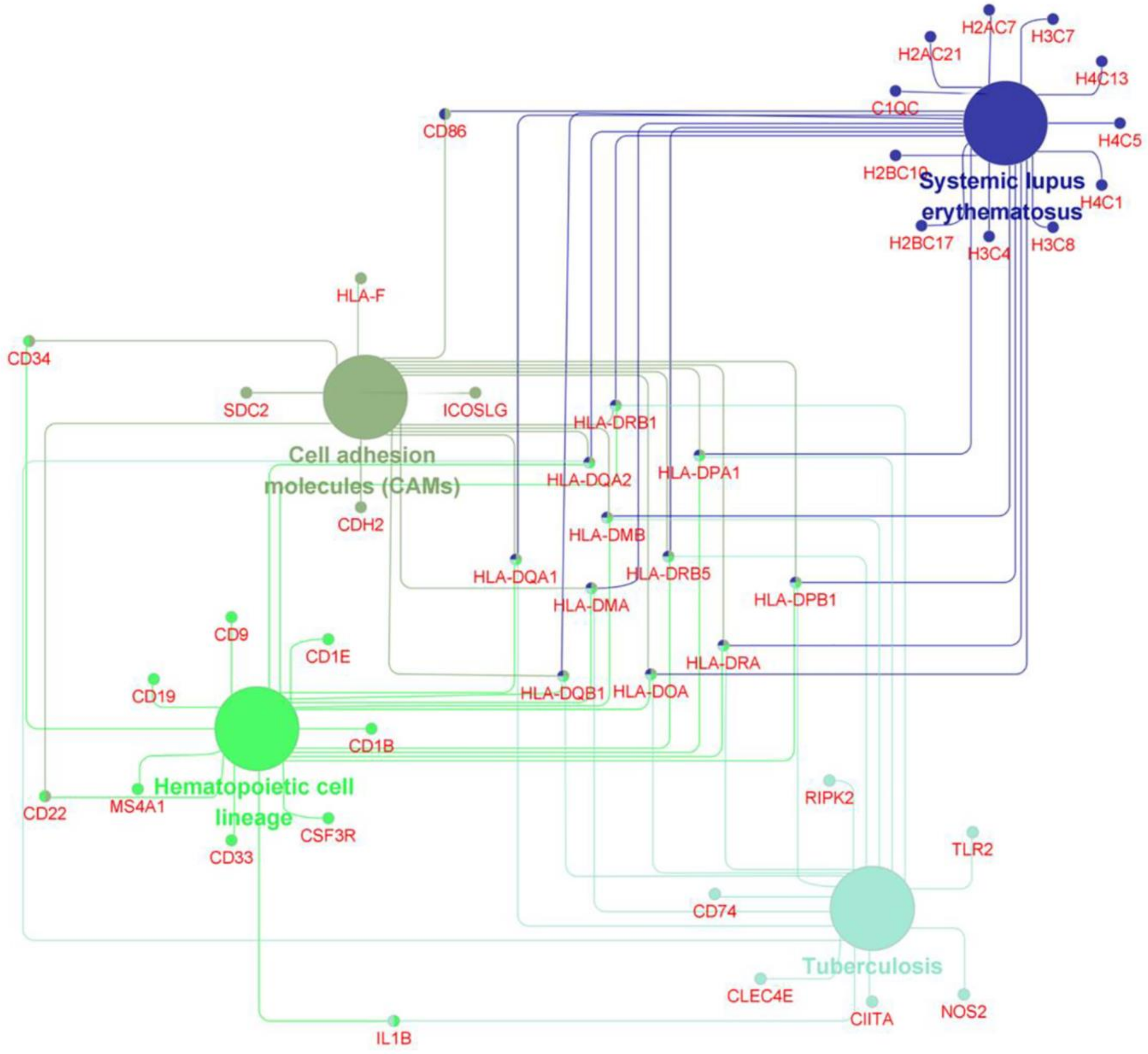

Supplementary Figure 4. Interrelation analysis was performed by assessing the KEGG pathway enrichment of the common DEGs.

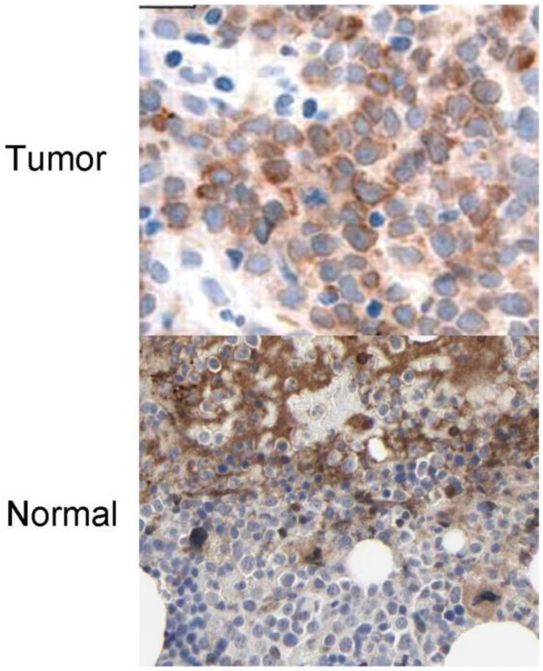

Supplementary Figure 5. Representative immunohistochemical pictures of ISCIRGs protein expression in BM of healthy and ALL patients, which was obtained from the Human Protein Atlas database and a research study (Rodig et al. The pre-B-cell receptor associated protein VpreB3 is a useful diagnostic marker for identifying c-MYC translocated lymphomas. https://doi.org/10.3324/haematol.2010.025767.) (only *VPREN3* has been found in present literatures and database).

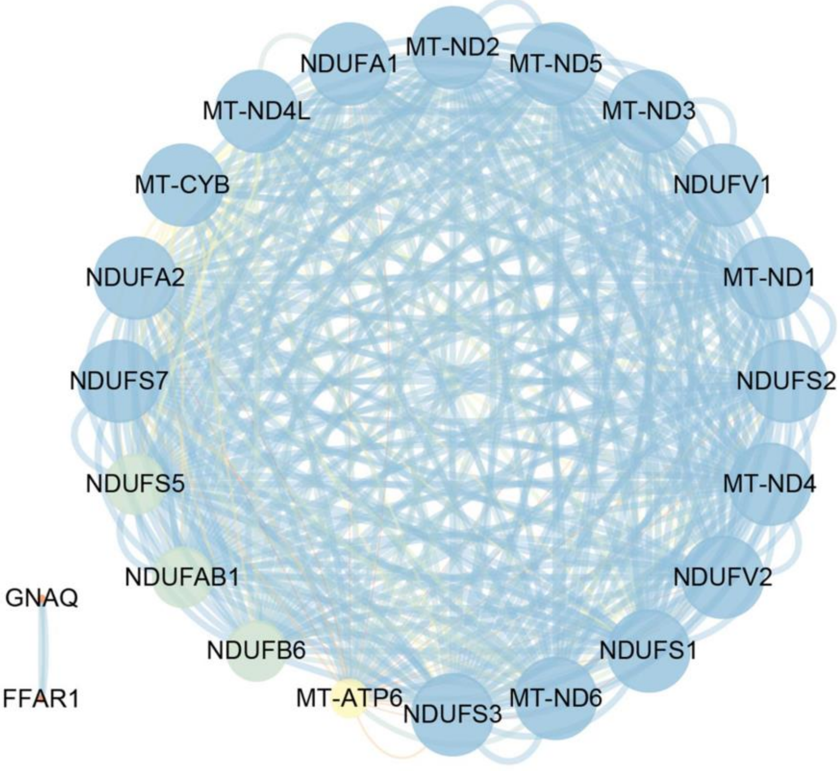

Supplementary Figure 6. PPI network for prognostic prediction DEGs. Note: The size of the node represents the degree, and the color of the node represents the *p*-value for prognosis. The warmer the color, the smaller the *p*-value, and the cooler the color, the greater the *p*-value.

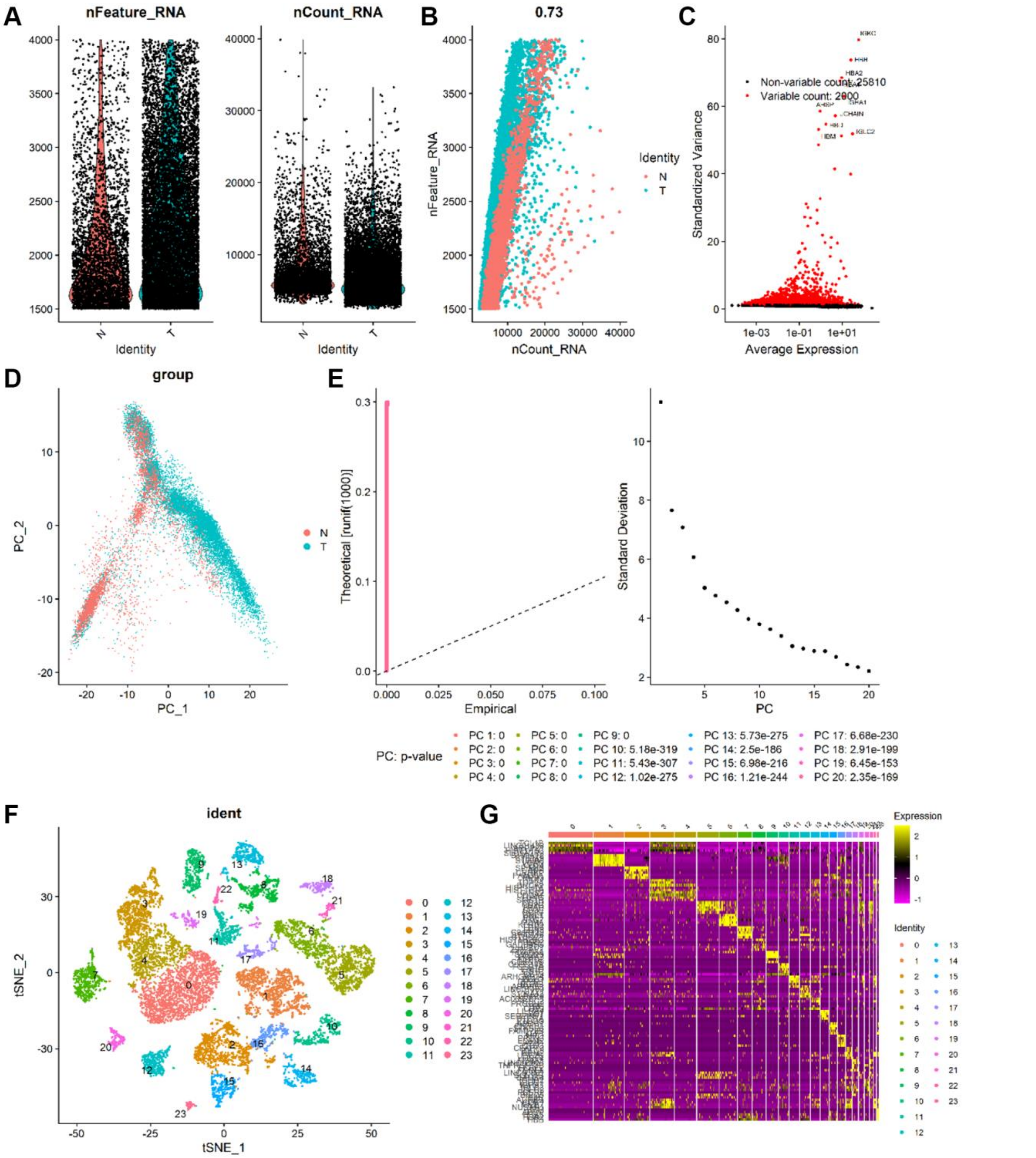

Supplementary Figure 7. Identification of 23 cell clusters with diverse annotations revealing high cellular heterogeneity in ALL based on single-cell RNA-seq data. (**A**) After quality control from the cores of 7 ALL and 4 non-malignant BM samples, 27,810 cells were included in the analysis. (**B**) The numbers of detected genes were significantly related to the sequencing depth, with a Pearson's correlation coefficient of 0.73. (**C**) The variance diagram shows 25,810 corresponding genes throughout all cells from GBMs. The red dots represent highly variable genes, and the black dots represent nonvariable genes. The top 10 most variable genes are marked in the plot. (**D**) PCA did not demonstrate clear separations of cells in GBMs. (**E**) PCA identified the 20 PCs with an estimated *p*-value <0.05. (**F**) The tSNE algorithm was applied for dimensionality reduction with the 20 PCs, and 23 cell clusters were successfully classified. (**G**) The differential analysis identified marker genes. The top 5 marker genes of each cell cluster are displayed in the heatmap. The colors from purple to yellow indicate the gene expression levels from low to high.

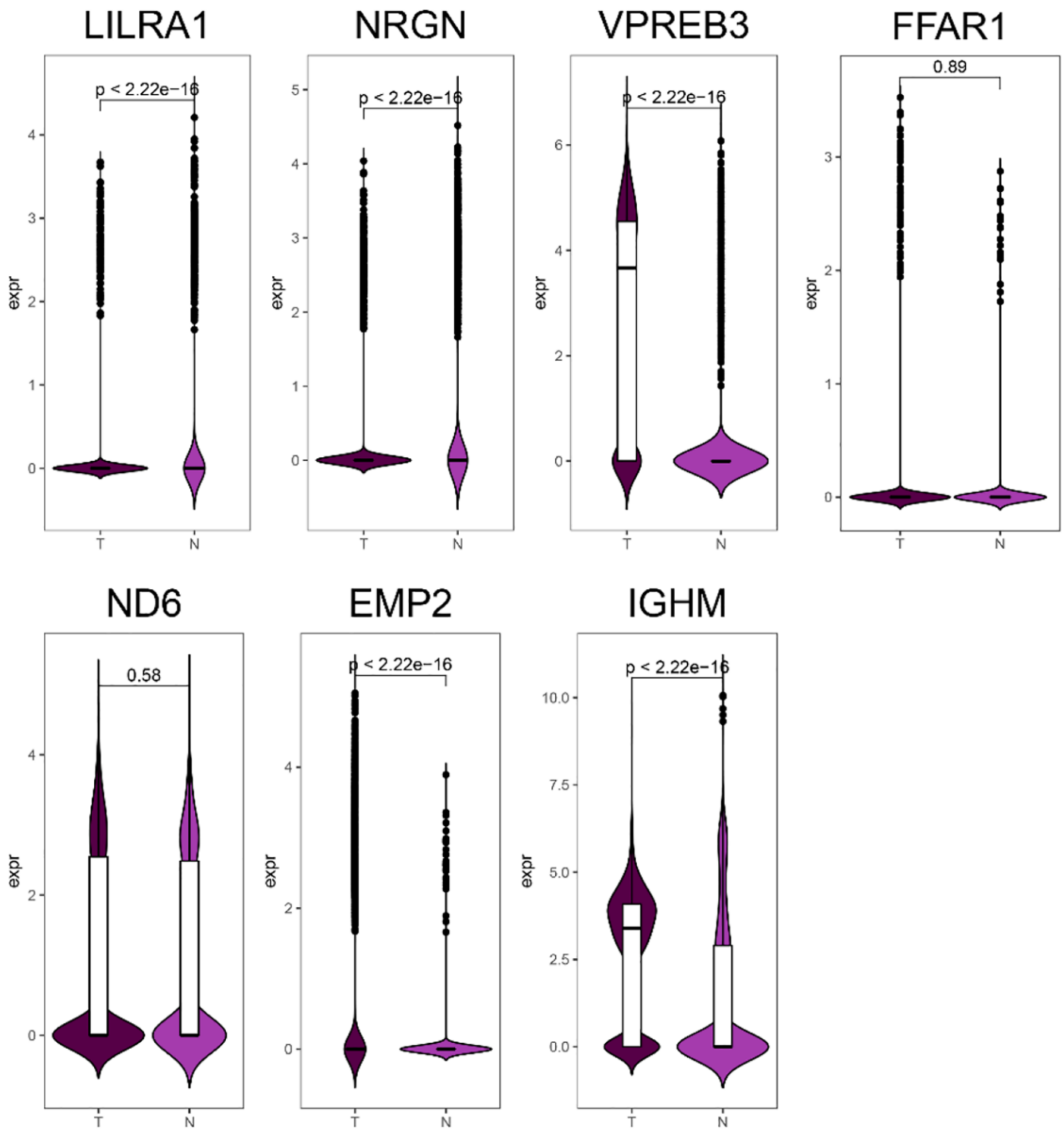

Supplementary Figure 8. The gene expression difference of the seven ISCIRGs between ALL and healthy BM based scRNA-seq datasets.

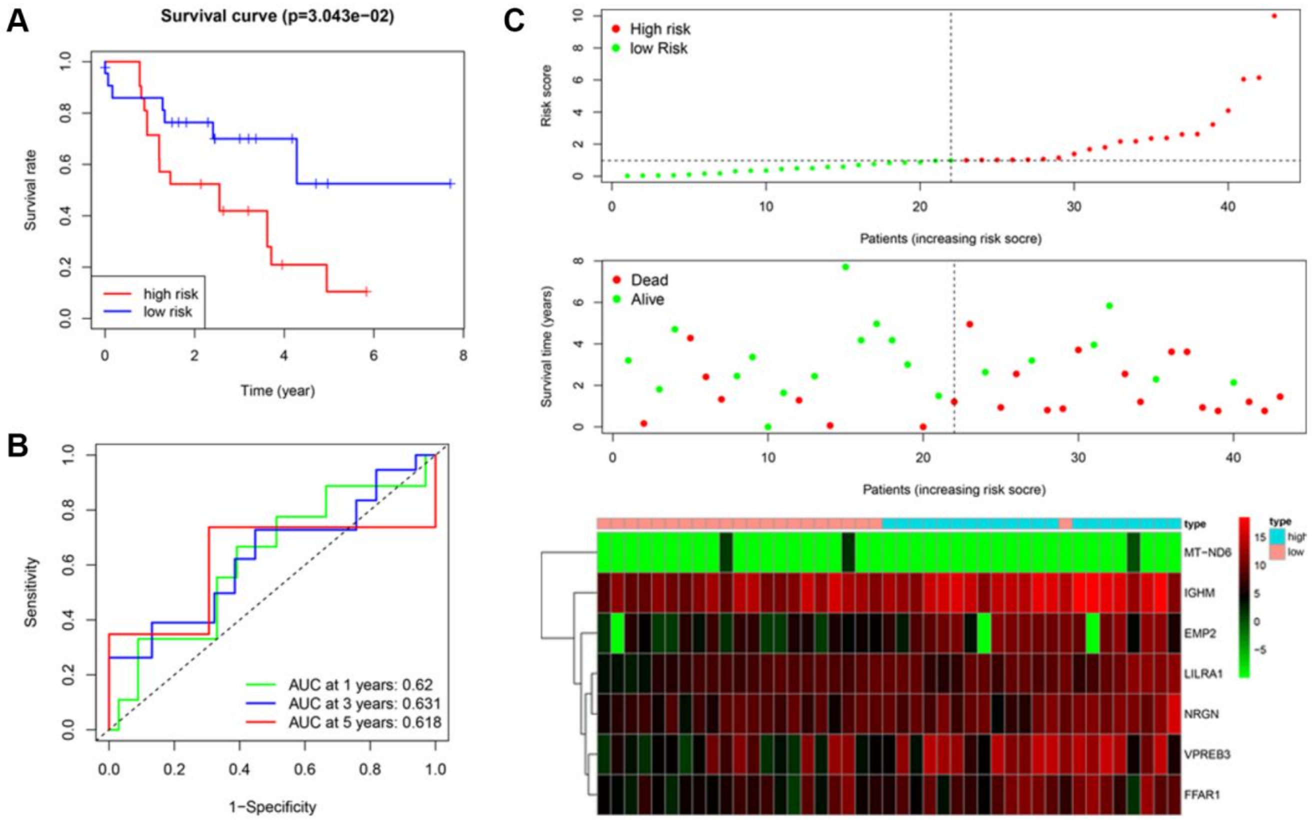

Supplementary Figure 9. Assessment of the prognostic value of the ISCIRG signature in validation group. (**A**) KM survival curve for high-risk and low-risk patients. (**B**) Time-dependent ROC curve for 1-, 3-, and 5-year OS rates. (**C**) Risk score analysis for the high-risk group and low-risk group. Upper panel: Patient survival status and time distributed by the risk score. Middle panel: Risk score curves of the ISCIRG signature. Bottom panel: Heatmaps of the expression levels of the seven ISCIRGs. The colors from green to red indicate the gene expression levels from low to high.

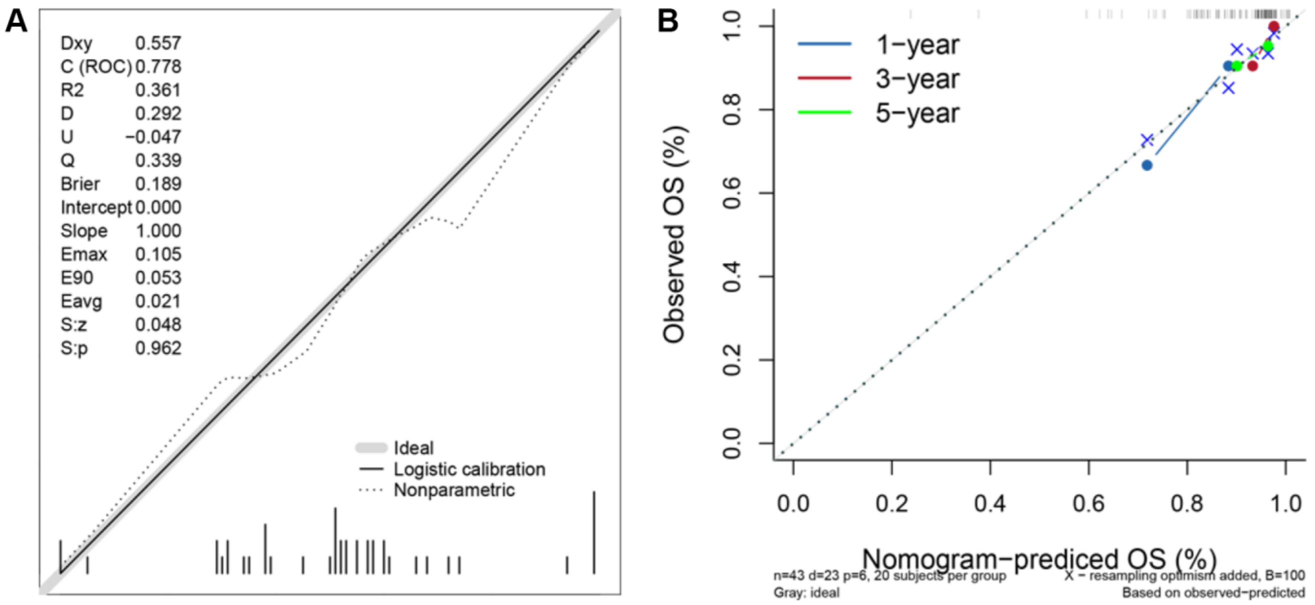

Supplementary Figure 10. Prognostic nomogram to predict the 1-, 3-, and 5-year OS in validation group. (**A**) Calibration test for the prognostic nomogram. (**B**) Calibration plot of the prognostic nomogram for predicting OS at 1, 3, and 5 years.

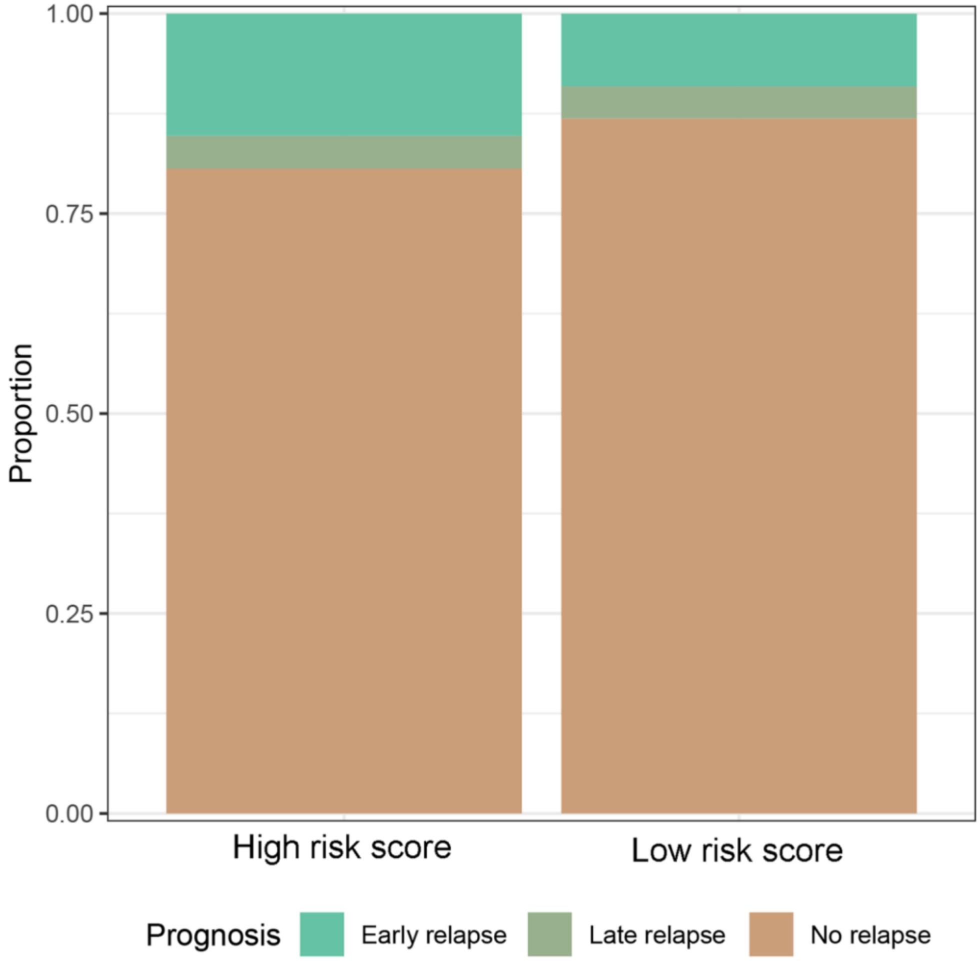

Supplementary Figure 11. Prognostic significance of risk score in external validation datasets (GSE13576).

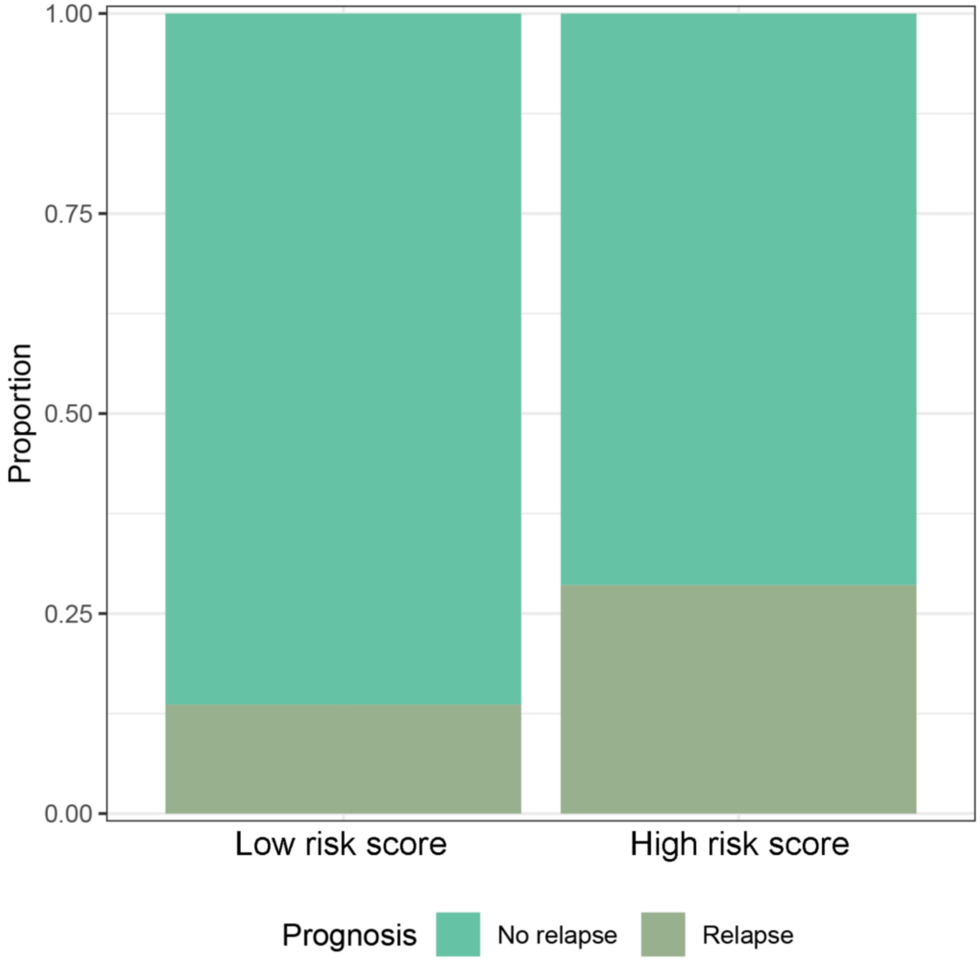

Supplementary Figure 12. Prognostic significance of risk score in external validation datasets (GSE50999).

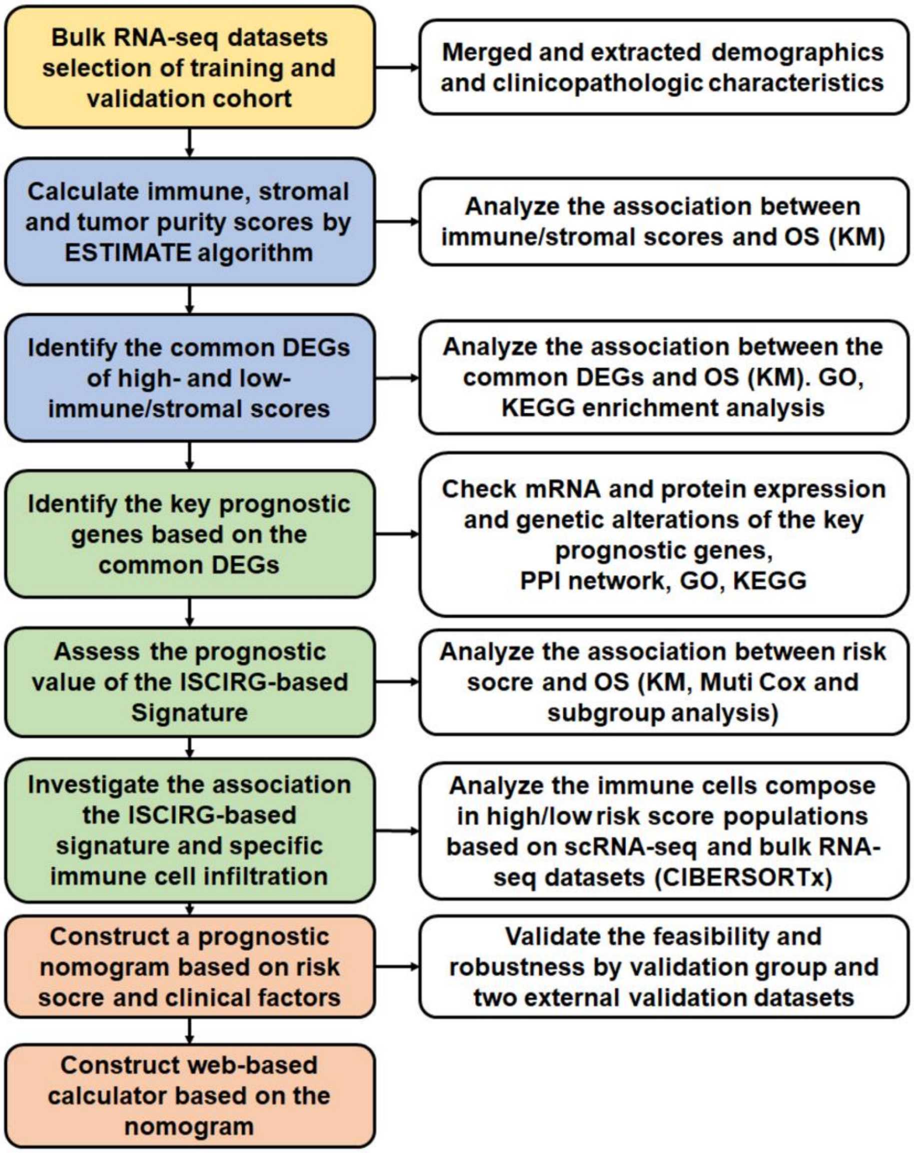

Supplementary Figure 13. The complete method design of our study.
