# Supplementary material for: Identification of immune and stromal cell infiltration-related gene signature for prognosis prediction in acute lymphoblastic leukemia
Source: Aging (Albany NY). 2022 Sep 19;14(18):7470–504. doi: 10.18632/aging.204292 (PMC9550239; doi:10.18632/aging.204292)
Supplement: Supplementary Table 2 [file aging-14-204292-s003.docx]

**Supplementary Table 2. KM survival analysis for all common DEGs.**

| **Gene** | **P-value** |
| --- | --- |
| HLA-DRB1 | 6.55E-12 |
| CD33 | 9.62E-07 |
| AC005077.4 | 6.65E-13 |
| MMP17 | 6.65E-11 |
| RNU6-1274P | 9.16E-09 |
| RIPK2 | 6.82E-12 |
| TCL1A | 1.54E-11 |
| NPY | 3.76E-10 |
| HLX | 2.71E-10 |
| LINC01374 | 2.05E-10 |
| MT-ATP8 | 3.42E-11 |
| PPP2R2C | 2.97E-13 |
| SERPING1 | 1.22E-09 |
| HLA-DQA2 | 3.44E-07 |
| LDLRAD2 | 1.02E-11 |
| BVES | 5.14E-14 |
| CIITA | 2.16E-11 |
| HLA-DRB6 | 2.02E-10 |
| GSN | 2.98E-11 |
| HBEGF | 1.20E-11 |
| LILRA2 | 5.38E-12 |
| AC107884.1 | 1.43E-10 |
| CDK14 | 1.84E-11 |
| AC134043.3 | 2.66E-15 |
| LY86 | 2.37E-07 |
| CALHM6 | 6.11E-08 |
| LINC02355 | 6.07E-09 |
| EBF1 | 6.77E-15 |
| C9orf139 | 0.000206 |
| KIR3DX1 | 7.44E-10 |
| ZC3H12D | 3.97E-13 |
| MS4A6A | 7.11E-05 |
| NGFR | 4.81E-09 |
| LILRA1 | 2.46E-10 |
| NAV1 | 1.61E-14 |
| TCL1B | 1.67E-15 |
| CD86 | 1.29E-05 |
| IGKV1OR2-108 | 2.78E-09 |
| CLECL1 | 1.29E-08 |
| MACROD2 | 2.95E-11 |
| JUP | 3.11E-09 |
| CD9 | 3.30E-12 |
| IQSEC2 | 3.64E-11 |
| C1QTNF4 | 5.23E-11 |
| AL133346.1 | 0 |
| PDE4B | 7.34E-08 |
| CD300LF | 0.00344 |
| NRGN | 5.97E-10 |
| MS4A14 | 0.000178 |
| PAX5 | 4.54E-12 |
| CXCL16 | 8.24E-12 |
| DIRAS1 | 3.28E-12 |
| MN1 | 1.39E-07 |
| EPHA7 | 9.96E-12 |
| GAPT | 1.78E-07 |
| SERPINB6 | 5.57E-09 |
| ALOX5 | 4.03E-08 |
| AC104237.2 | 9.36E-08 |
| CEACAM6 | 0.003656 |
| RBM47 | 5.52E-07 |
| IRAK3 | 2.90E-08 |
| LINC01480 | 1.98E-11 |
| LINC02202 | 2.55E-14 |
| TENM4 | 3.36E-11 |
| PTGS1 | 4.74E-07 |
| TNNI2 | 6.62E-08 |
| MS4A7 | 0.002826 |
| NCF4 | 1.73E-12 |
| RHBDF2 | 4.05E-11 |
| HLA-DQB1 | 2.50E-10 |
| HLA-DQB1-AS1 | 3.25E-10 |
| TNS3 | 4.84E-10 |
| PLVAP | 1.14E-09 |
| MEF2C-AS1 | 3.59E-10 |
| PANX2 | 1.83E-11 |
| VPREB3 | 3.17E-13 |
| CYTL1 | 1.48E-07 |
| TRERNA1 | 9.99E-16 |
| AL590226.1 | 1.53E-11 |
| TP53I11 | 2.87E-10 |
| BTK | 9.44E-12 |
| AC124248.1 | 7.85E-13 |
| AC092490.3 | 2.20E-10 |
| HLA-DPA1 | 3.08E-11 |
| SDC2 | 1.03E-12 |
| EGFL7 | 1.16E-08 |
| DIPK1C | 2.43E-07 |
| SLC2A5 | 6.27E-12 |
| WFS1 | 2.20E-10 |
| TMEM273 | 2.73E-07 |
| LINC00958 | 1.11E-11 |
| DUSP26 | 3.35E-12 |
| ECM1 | 1.44E-15 |
| U62631.1 | 8.13E-13 |
| LINC01922 | 2.47E-12 |
| BLNK | 3.26E-12 |
| IGHD | 7.08E-12 |
| ELOCP28 | 5.91E-11 |
| THEMIS2 | 1.38E-11 |
| SLC9A3R2 | 5.78E-12 |
| OVCH2 | 7.88E-11 |
| NPR1 | 3.68E-11 |
| HLA-DRA | 7.77E-11 |
| MT-ND6 | 1.21E-11 |
| C1QC | 3.10E-07 |
| HLA-DMA | 2.80E-11 |
| LINC01150 | 2.91E-09 |
| CD74 | 1.64E-11 |
| MT-ND4 | 1.01E-11 |
| ROBO4 | 7.41E-07 |
| OGFRL1 | 7.31E-11 |
| BANK1 | 1.32E-10 |
| ICOSLG | 2.21E-13 |
| ZNF296 | 1.76E-12 |
| CYGB | 2.36E-11 |
| TBXA2R | 1.18E-12 |
| LINC02422 | 3.64E-09 |
| NOS2 | 5.05E-11 |
| AC008060.4 | 1.42E-11 |
| LILRB1 | 3.44E-11 |
| STK32B | 2.46E-10 |
| OR5P3 | 1.70E-08 |
| SERPINI2 | 1.58E-09 |
| HTRA3 | 2.31E-12 |
| GIPC3 | 3.79E-13 |
| CD34 | 1.11E-07 |
| PDLIM1 | 3.08E-12 |
| AC125603.1 | 1.19E-05 |
| KCNA5 | 2.56E-12 |
| HLA-F | 1.20E-10 |
| S100A16 | 5.35E-13 |
| GNG7 | 2.09E-11 |
| HLA-DRB5 | 5.85E-12 |
| KLF11 | 3.85E-07 |
| AC136424.2 | 6.06E-14 |
| LRP3 | 1.08E-08 |
| AC025437.5 | 2.33E-15 |
| SPATC1 | 2.75E-09 |
| CYRIA | 6.96E-10 |
| RASAL2 | 5.47E-09 |
| CYBB | 0.000667 |
| ZNF467 | 1.95E-09 |
| SDK2 | 1.19E-07 |
| AC027348.1 | 9.99E-16 |
| IL1B | 8.48E-10 |
| LINC01013 | 1.23E-13 |
| AL139020.1 | 6.22E-15 |
| CSF3R | 5.73E-06 |
| HLA-DOA | 2.14E-11 |
| CD19 | 4.00E-12 |
| JCHAIN | 4.05E-05 |
| BLACE | 2.45E-13 |
| VENTX | 6.72E-09 |
| CRMP1 | 5.42E-13 |
| AC099524.1 | 8.49E-14 |
| PLD4 | 2.34E-07 |
| LPCAT2 | 6.32E-05 |
| ADCY9 | 3.23E-11 |
| CLEC4E | 7.57E-07 |
| GNAI1 | 8.01E-11 |
| FUT7 | 0.00279 |
| BCL3 | 1.17E-10 |
| LILRB4 | 9.82E-06 |
| CD22 | 8.37E-12 |
| HLA-DQB2 | 3.81E-11 |
| CHST15 | 1.18E-11 |
| GNG11 | 2.53E-08 |
| LAMC1 | 2.44E-11 |
| HLA-DQA1 | 4.13E-12 |
| AL713998.1 | 8.88E-16 |
| TCL6 | 1.00E-10 |
| LINC02413 | 2.22E-16 |
| RIN2 | 2.46E-08 |
| EMP2 | 3.99E-13 |
| NCF1C | 7.95E-12 |
| LARGE1 | 1.81E-11 |
| ZNF703 | 7.69E-11 |
| PSD3 | 6.98E-14 |
| CORO2B | 1.65E-13 |
| AC125603.2 | 0.000203 |
| AL021408.1 | 3.29E-14 |
| AC133065.3 | 2.91E-12 |
| AC092490.2 | 9.55E-12 |
| LTBR | 1.77E-10 |
| IGHM | 2.46E-11 |
| LCN8 | 2.62E-13 |
| CYP2C8 | 2.75E-11 |
| MS4A4A | 0.022141 |
| CRIM1-DT | 5.80E-12 |
| ADGRD1 | 1.63E-10 |
| RPL32P1 | 8.93E-11 |
| LDOC1 | 1.90E-10 |
| TNFSF12 | 9.67E-11 |
| TYROBP | 7.82E-07 |
| BMP2 | 1.16E-12 |
| AC087627.1 | 4.71E-10 |
| AC008060.1 | 6.50E-11 |
| LILRB2 | 2.52E-12 |
| SCHIP1 | 1.11E-05 |
| SH2D4B | 9.88E-12 |
| HLA-DMB | 3.09E-11 |
| MS4A4E | 0.012977 |
| AC015660.4 | 9.99E-16 |
| LCN6 | 2.78E-15 |
| AC006538.3 | 1.48E-14 |
| CYSLTR1 | 1.92E-06 |
| IL13RA1 | 5.95E-11 |
| CCN2 | 4.53E-13 |
| TLR2 | 3.84E-09 |
| AC022182.2 | 1.28E-10 |
| LINC00968 | 3.87E-10 |
| AC005229.3 | 5.13E-12 |
| TLR10 | 4.62E-11 |
| KLF2 | 1.76E-11 |
| HGF | 1.37E-10 |
| HHEX | 2.70E-09 |
| FOLR2 | 2.83E-09 |
| AC008957.1 | 2.08E-12 |
| TMPRSS15 | 9.19E-09 |
| HLA-DPB1 | 3.40E-11 |
| AC104237.3 | 4.86E-10 |
| MEF2C | 1.18E-10 |
| SPI1 | 1.69E-11 |
| CYB5R2 | 9.10E-08 |
| LINC01747 | 8.43E-13 |
| CLEC14A | 2.58E-13 |
| LINC00114 | 3.33E-16 |
| IGHJ3P | 6.70E-09 |
| RAB17 | 1.31E-12 |
| MS4A1 | 1.66E-09 |
| LRRK1 | 1.38E-10 |
| ITPRIPL2 | 0.000492 |
| FFAR1 | 6.12E-12 |
| TUSC3 | 0.000334 |
| RN7SKP185 | 2.27E-12 |
| MIR137HG | 5.01E-12 |
| PEX5L-AS2 | 6.42E-13 |
| AJ011931.1 | 3.63E-11 |
| AC105150.1 | 1.72E-10 |
| AJAP1 | 1.61E-05 |
| ADGRB2 | 4.00E-05 |
| IGHV3-19 | 5.63E-13 |
| SNORD17 | 2.28E-10 |
| RN7SKP79 | 1.72E-11 |
| RN7SKP8 | 3.23E-12 |
| RN7SKP118 | 2.57E-13 |
| PCDH10 | 3.97E-09 |
| AL133492.1 | 1.60E-13 |
| AL138899.2 | 7.65E-10 |
| RN7SL674P | 2.41E-11 |
| RMRP | 1.13E-07 |
| CD1B | 5.63E-13 |
| CHRNA3 | 6.03E-10 |
| H2AC7 | 1.17E-09 |
| NOTCH3 | 3.27E-06 |
| ILDR2 | 7.19E-11 |
| H4C5 | 9.68E-11 |
| RN7SKP291 | 4.07E-12 |
| SCARNA13 | 1.19E-09 |
| SNORA74A | 6.48E-09 |
| AC010275.1 | 5.63E-11 |
| RN7SL5P | 7.73E-11 |
| IGHV3-35 | 1.17E-12 |
| RN7SKP255 | 7.87E-13 |
| RNF150 | 3.60E-10 |
| MKRN3 | 4.30E-07 |
| RN7SL396P | 4.26E-10 |
| AC247036.1 | 3.93E-12 |
| LINC00029 | 1.09E-10 |
| LINC01882 | 2.72E-12 |
| AL133368.1 | 1.39E-10 |
| ALDH1A2 | 5.24E-06 |
| H2BC10 | 6.95E-11 |
| H3C7 | 5.32E-11 |
| AL365440.1 | 1.21E-08 |
| SNORA49 | 1.70E-08 |
| SPSB4 | 8.75E-10 |
| CDH2 | 2.52E-05 |
| AL163932.1 | 2.30E-09 |
| RN7SKP80 | 1.44E-12 |
| IGHV3-37 | 4.90E-12 |
| SNORA47 | 2.65E-10 |
| RN7SKP9 | 4.00E-11 |
| ENPP7P11 | 7.29E-13 |
| LINC01120 | 1.99E-10 |
| SLIT1 | 1.89E-12 |
| PEX5L-AS1 | 2.74E-13 |
| AJ239328.1 | 6.19E-11 |
| H2AC21 | 1.51E-07 |
| BHLHB9P1 | 6.26E-11 |
| PCBP3-AS1 | 3.26E-11 |
| PPP1R1C | 3.89E-12 |
| H4C13 | 4.79E-12 |
| GPR87 | 1.34E-10 |
| PABPC4L | 6.82E-07 |
| AC124312.3 | 1.25E-08 |
| H1-5 | 2.20E-08 |
| H1-4 | 1.33E-07 |
| AC073370.1 | 1.30E-11 |
| IGHV3-33-2 | 6.28E-11 |
| RN7SKP90 | 7.43E-11 |
| PTCRA | 1.18E-06 |
| RN7SKP71 | 4.53E-12 |
| ARHGAP19-SLIT1 | 7.34E-12 |
| GXYLT2 | 1.52E-13 |
| AL138899.1 | 8.18E-09 |
| AC084082.1 | 3.45E-10 |
| RN7SKP217 | 1.25E-09 |
| SNORA73B | 3.19E-10 |
| NDST3 | 3.07E-11 |
| RN7SK | 6.33E-10 |
| GLP1R | 3.99E-07 |
| RN7SL4P | 4.78E-12 |
| SHISAL1 | 2.50E-06 |
| ELOVL4 | 1.37E-12 |
| IGHV3-36 | 1.62E-11 |
| RN7SKP48 | 3.38E-13 |
| POF1B | 5.40E-09 |
| SCARNA21 | 8.95E-10 |
| KRT18P29 | 6.13E-10 |
| RN7SKP203 | 1.46E-13 |
| PEX5L | 7.87E-13 |
| PPIAP52 | 1.26E-08 |
| SCARNA5 | 3.04E-11 |
| H4C1 | 3.46E-12 |
| H2BC17 | 4.23E-11 |
| H3C8 | 1.16E-09 |
| IGHV7-34-1 | 5.44E-12 |
| SNORD116-19 | 1.57E-11 |
| CD1E | 8.44E-12 |
| H3C4 | 5.99E-12 |
| H1-3 | 6.84E-08 |
| SCARNA7 | 8.60E-10 |
| FAT1 | 0.02296 |
| LCT-AS1 | 2.04E-10 |
