# Supplementary material for: Identification of immune and stromal cell infiltration-related gene signature for prognosis prediction in acute lymphoblastic leukemia
Source: Aging (Albany NY). 2022 Sep 19;14(18):7470–504. doi: 10.18632/aging.204292 (PMC9550239; doi:10.18632/aging.204292)
Supplement: Supplementary Tables 3-4 and 7 [file aging-14-204292-s004.pdf]

SUPPLEMENTARY TABLES

Supplementary Table 3. Significant OS-predicting ISCIRGs.

Gene	coef	HR	HR.95L	HR.95H	*P*-value
LILRA1	0.001077	1.001078	1.000569	1.001587	3.29E-05
NRGN	0.000137	1.000137	0.999987	1.000287	0.073113
VPREB3	0.000019	1.000019	1.000001	1.000036	0.036097
MT-ND6	0.000005	1.000005	1.000002	1.000009	0.001497
EMP2	0.000259	1.000259	0.999991	1.000528	0.05815
IGHM	0.000003	1.000003	1	1.000007	0.061815
FFAR1	0.000133	1.000133	1.000057	1.000209	0.000649

Supplementary Table 4. GO and KEGG pathway enrichment for the seven OS-predicting ISCIRGs.

Term ID	Term description	Observed gene count	Background gene count	Strength
BP				
GO:0006120	Mitochondrial electron transport, nadh to ubiquinone	18	49	2.44
GO:0006119	Oxidative phosphorylation	20	118	2.11
GO:0042775	Mitochondrial atp synthesis coupled electron transport	19	87	2.22
GO:0032981	Mitochondrial respiratory chain complex i assembly	17	66	2.29
GO:0007005	Mitochondrion organization	18	452	1.48
GO:0022607	Cellular component assembly	18	2359	0.76
GO:0015990	Electron transport coupled proton transport	4	5	2.78
GO:0006996	Organelle organization	19	3450	0.62
GO:1902600	Proton transmembrane transport	5	150	1.4
MF				
GO:0008137	NADH dehydrogenase (ubiquinone) activity	18	46	2.47
GO:0016491	Oxidoreductase activity	19	726	1.29
GO:0051536	Iron-sulfur cluster binding	5	66	1.76
GO:0009055	Electron transfer activity	5	103	1.56
GO:0003824	Catalytic activity	20	5486	0.44
GO:0051539	4 iron, 4 sulfur cluster binding	4	42	1.86
GO:0048038	Quinone binding	3	18	2.1
CC				
GO:0070469	Respirasome	19	95	2.18
GO:0005747	Mitochondrial respiratory chain complex i	17	48	2.43
GO:0098803	Respiratory chain complex	18	80	2.23
GO:0005746	Mitochondrial respirasome	18	84	2.21
GO:0098800	Inner mitochondrial membrane protein complex	19	131	2.04
GO:1990204	Oxidoreductase complex	18	107	2.1
GO:0005743	Mitochondrial inner membrane	20	480	1.5
GO:0031966	Mitochondrial membrane	21	722	1.34
GO:0031967	Organelle envelope	22	1213	1.14

GO:0098796	Membrane protein complex	20	1141	1.12
GO:1902494	Catalytic complex	19	1328	1.03
GO:0031090	Organelle membrane	23	3548	0.69
GO:0016020	Membrane	25	9072	0.32
GO:0043231	Intracellular membrane-bounded organelle	24	10761	0.22
GO:0005737	Cytoplasm	24	11428	0.2
GO:0005759	Mitochondrial matrix	5	479	0.9
KEGG				
hsa00190	**Oxidative phosphorylation**	20	130	2.06
hsa04723	Retrograde endocannabinoid signaling	19	145	1.99
hsa05016	Huntington disease	21	298	1.72
hsa04714	Thermogenesis	20	229	1.82
hsa05012	Parkinson disease	20	240	1.8
hsa05020	Prion disease	20	265	1.75
hsa05010	Alzheimer disease	21	355	1.65
hsa05014	Amyotrophic lateral sclerosis	20	352	1.63
hsa04932	Non-alcoholic fatty liver disease	12	148	1.79
hsa01100	Metabolic pathways	19	1447	0.99

Supplementary Table 7. Broad cell type markers.

Cell type	Genes
B cells	BLK, BLNK, CD19, CD72, CD79A, IGHA1, IGHG1, IGKC, IGL, PAX5
Erythrocytic	ALAS2, HBA1, HBA2, HBQ1, RHCE
HSPCs	ATP8B4, CLEC3B, CRHBP, CRYGD, ELN, EXD2, FAM124B, FLT3, HLF, HOXA3, HOXB6, KCNJ13, KLF1, LAPTM4B, MEIS1, MMRN1, MPL, SPINK2, THY1, VWF
Myeloid cells	ATG7, BST1, CCL7, CD14, CD163, CD1C, CD68, CD83, CD93, CEACAM3, CEBPA, CHI3L1, CHIT1, CLEC5A, COL8A2, CREB5, CRISPLD2, CSF1R, CSF3R, CTSK, CXCL5, CYBB, DNASE2B, DYSF, EMP1, FCAR, FCGR3A, FCGR3B, FLT3, FPR1, FPR2, FUT4, G0S2, GM2A, HLA-DRA, ITGAM, LILRB2, MARCO, ME1, MS4A4A, MSR1, PCOLCE2, PDE4B, PTGDS, S100A12, SCARB2, SIGLEC3, SIGLEC5, SPI1, SULT1C2, TNFRSF10C, VNN3
NK cells	CASP5, GSG1, IL18RAP, KLRB1, KLRD1, L1TD1, NCAM1, NCR1, NMUR1, PTGDR, TBX21, TP53TG5, XCL1
T cells	BCL11B, CD2, CD27, CD28, CD3D, CD3G, LAT
DCs	CD1a, CD40, CD80, CD86, MHC class II, CD11c, CD1a, CD1c, CD206, CD209, S100A8, S100A9, SIRPA, BDCA1, BTLA, CADM1, CD141, CD226, CD26, CLEC9A, DNAM-1, XCR1, CD11b, CD2, CLEC10A, CLEC4A, DCIR, Fc-epsilon RI-alpha, ILT1, SIRPA, BDCA2, BDCA-4, CD123, CD303, CD304, CLEC4C, DR6, Fc-epsilon RI-alpha, ILT3, ILT7, NRP1, CLEC10A, CLEC4A
